# *Borrelia burgdorferi* disrupts gut barrier functions and microbiota–antibody interactions

**DOI:** 10.1084/jem.20252380

**Published:** 2026-08-24

**Authors:** Shilpa Sachan, Giang Vu Vi Tran, Kimberly J. Olsen, Heather Kulaga, Anne Day, Hannah P. Savage, Alison W. Rebman, John N. Aucott, Isabel A. Jimenez, Cory F. Brayton, Nicole Baumgarth

**Affiliations:** 1 W. Harry Feinstone Department of Molecular Microbiology and Immunology, Lyme and Tickborne Diseases Research and Education Institute, Johns Hopkins Bloomberg School of Public Health, Johns Hopkins University, Baltimore, MD, USA; 2 Center for Immunology and Infectious Diseases and Department of Pathology, Microbiology and Immunology, School of Veterinary Medicine, University of California, Davis, CA, USA; 3 Department of Microbiology and Immunology, School of Medicine, University of California, Davis, CA, USA; 4 Lyme Disease Research Center, Division of Rheumatology, Department of Medicine, Johns Hopkins University School of Medicine, Baltimore, MD, USA; 5 Department of Molecular and Comparative Pathobiology, Johns Hopkins University School of Medicine, Baltimore, MD, USA

## Abstract

Lyme disease, an emerging tickborne disease caused by *Borrelia burgdorferi* (Bb), can result in myriad symptoms of unknown causes in humans. Here, we show that Bb rapidly disrupts the gastrointestinal immune barrier in infected mice, inducing a “leaky gut” syndrome, characterized by increased gut permeability, systemic endotoxemia, elevated LPS binding protein and intestinal fatty acid binding protein, and altered blood leukocyte profiles. Patients with acute Lyme disease showed similar blood changes that correlated with disease symptoms and liver function measurements and largely resolved after antibiotic treatment. Mechanistically, Bb infection triggered mild early inflammation in the small intestine and suppressed humoral immunity to the microbiota, resulting in reduced IgA coating of fecal microbes and decreased antimicrobial serum IgG, despite the increased gut permeability. These defects preceded shifts in later observed gut bacterial composition and short-chain fatty acid production. Together, our findings identify mucosal barrier dysfunction and impaired antimicrobial humoral immunity as potential drivers of Bb-induced disease.

## Teaser

Dysregulated immune responses rather than damage by *Borrelia burgdorferi* alone induce Lyme disease, but mechanisms of disease are poorly understood. Sachan et al. demonstrate that infected mice rapidly develop gut leakiness, endotoxemia, reduced microbiota-reactive antibodies, and systemic markers of inflammatory, some also seen in human Lyme disease patients.

## Graphical Abstract

**Figure:**
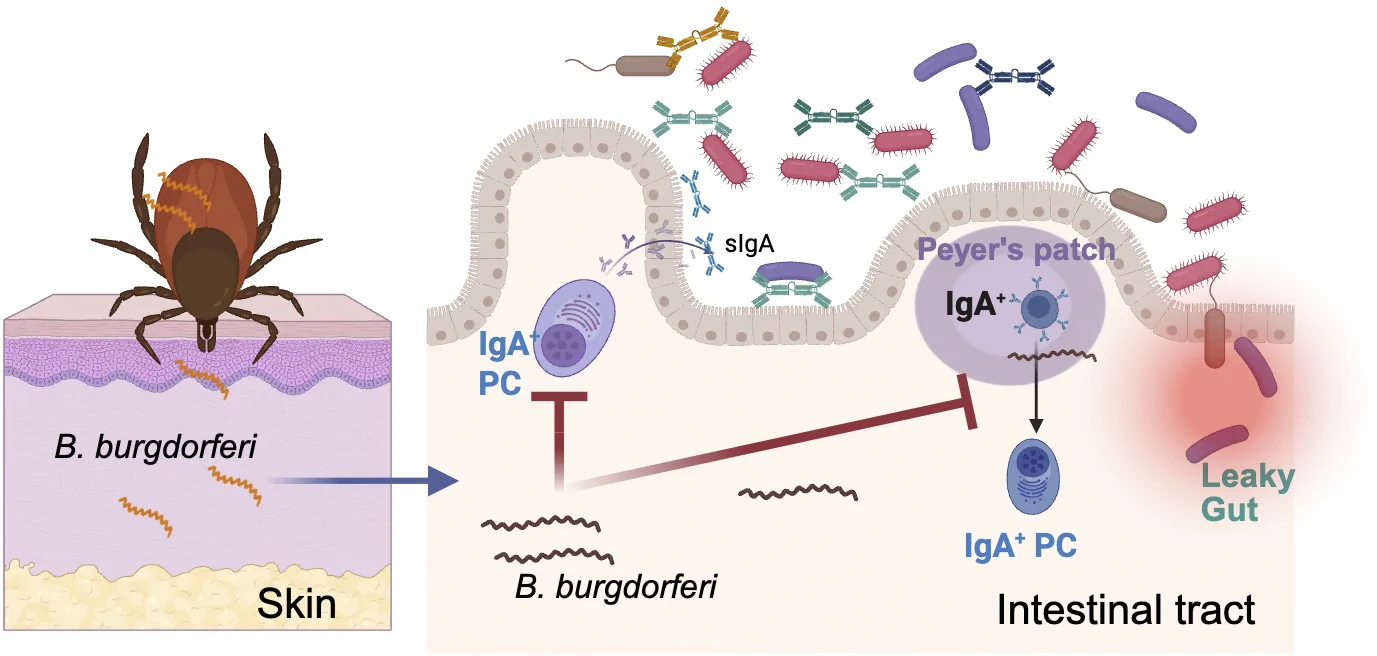

## Introduction

Lyme disease, caused by the spirochete bacterium *Borrelia burgdorferi* (Bb) and transmitted by infected ticks, is the most common vector-borne illness in the Northern Hemisphere with an estimated 476,000 cases diagnosed and treated annually in the United States alone (Bobe et al., 2021). With tick populations expanding geographically, Lyme disease is emerging as an increasingly significant public health threat. Early symptoms of infections in humans typically include an expanding red skin rash (erythema migrans), fever, headache, and fatigue (Steere et al., 1986). When left untreated, the infection can disseminate, leading to severe manifestations of inflammation affecting various organ systems, including the joints (Lyme arthritis), nervous system (neuroborreliosis), and heart (Lyme carditis) (Strle and Stanek, 2009). A significant concern is posttreatment Lyme disease syndrome (PTLDS), where symptoms such as fatigue, pain, gastrointestinal (GI) disturbances, and cognitive dysfunction persist after antibiotic treatment, similar to observations in “Long COVID” (Aucott et al., 2022; Rebman and Aucott, 2020; Yong, 2021). Large-scale proteomic studies reported that PTLDS patients fail to return to preinfection steady state with increases in serum biomarkers related to acute-phase proteins/liver functions, metabolic changes, and ongoing innate immune system changes, even long after completion of treatment (Fitzgerald et al., 2021; Zhou et al., 2019).

Bb is well known for its persistence in host tissues of their native reservoir hosts, a variety of rodents and small birds, as well as deer (Piesman and Gern, 2004). In laboratory mice, Bb was shown to evade and suppress the host immune system (Elsner et al., 2015; Hastey et al., 2012; Tunev et al., 2011; Williams et al., 2024). Our previous studies demonstrated that Bb infection in mice leads to rapid contraction of germinal centers (GCs) and a failure to induce effective humoral immunity (Elsner et al., 2015; Hastey et al., 2012). These effects extend beyond the primary infection site and agent, significantly dampening humoral immunity to unrelated antigens, for example, following immunization with influenza virus in alum and with SARS-CoV-2 spike protein (Elsner et al., 2015; Williams et al., 2024). Notably, even after antibiotic treatment, infected mice failed to develop robust immunological memory, suggesting that Bb not only disrupts the ongoing immune response but may also impair the generation of protective long-lived plasma cells and memory B cells, crucial for sustained host defense (Elsner et al., 2015; Tracy and Baumgarth, 2017).

The GI immune system orchestrates a delicate balance to maintain a functional epithelial barrier that protects the host from invasion by luminal microbiota, while also inducing tolerance to food antigens and mounting effective humoral and cellular defenses against invading pathogens (Kogut, 2022; Di Sabatino et al., 2023). In the steady state, the intestinal barrier maintains selective permeability, allowing essential nutrients and ions to pass, while preventing the translocation of harmful macromolecules. Disruption of this barrier, commonly referred to as “leaky gut” or increased intestinal permeability, allows microbial products to activate local and systemic inflammation (Vancamelbeke and Vermeire, 2017). Persistent inflammation can, in turn, contribute to the development and progression of chronic diseases (Takiishi et al., 2017). The GI microbiota also shapes B cell development, activation, and function, particularly through the induction of IgA- and IgG-producing B cells that are vital for maintaining mucosal immunity and a healthy microbial balance (Seikrit and Pabst, 2021; Yu et al., 2021). Disruption of this intricate interaction directly impairs the host’s ability to control and shape the microbial community (Christovich and Luo, 2022). This can lead to altered immune responses and significant changes to the gut microbial composition (Yoo et al., 2020). Through the “gut–brain axis,” such changes may also result in neurological and behavioral changes (Mayer et al., 2015; Osadchiy et al., 2019).

Indeed, gut dysbiosis has been implicated in the pathogenesis and severity of several chronic conditions, including post-COVID syndrome (Moreno-Corona et al., 2023), chronic fatigue syndrome (König et al., 2022), and autoimmune diseases like rheumatoid arthritis (Attur et al., 2022). A previous study on patients with PTLDS suggested that these patients harbor distinct gut microbiome signatures compared with both healthy individuals and intensive care unit patients (Morrissette et al., 2020). However, the extent to which Bb infection induced changes to the microbial communities on mucosal surfaces and the mechanisms underlying such potential changes are currently unknown.

This study reveals that Bb infection of mice rapidly disrupts the mucosal gut barrier in an apparent stepwise process, in which mild GI inflammation followed by reductions of local IgA and systemic IgG production diminishes protection from microbial invasion, resulting in microbiota-induced endotoxemia and other signs of systemic inflammation that preceded changes to the microbiota itself. Serum of patients presenting with acute Lyme disease showed similar signs of systemic inflammation, which were associated with the number of acute disease symptoms and liver function measurements, and which returned to levels seen in healthy controls (HCs) in patients 6 months after antibiotic treatment.

## Results

### Bb colonizes the GI tract and elicits an early, mild local inflammatory response

In mice, Bb disseminates rapidly from the site of infection and migrates to various distal tissues (Hyde, 2017; Tunev et al., 2011). Although the spirochete’s ability to evade host immunity is well documented, specific aspects of its impact on the adaptive immune response are particularly notable. Previous work showed that Bb is associated with the suppression of B cell responses to Bb and other coadministered antigens, a collapse of GCs, and a lack of durable immunological memory. This waning of functional memory becomes particularly evident when antibiotic treatment is administered, leading to a rapid decline in Bb-specific antibodies and susceptibility to reinfection (Elsner et al., 2015; Hastey et al., 2012; Piesman et al., 1997). Here, we sought to investigate the impact of Bb infection on mucosal barrier functions, focusing on the GI tract, as an intact immune system is critical for the regulation of the local microbiome and host–microbiome interactions.

**Figure 1.**
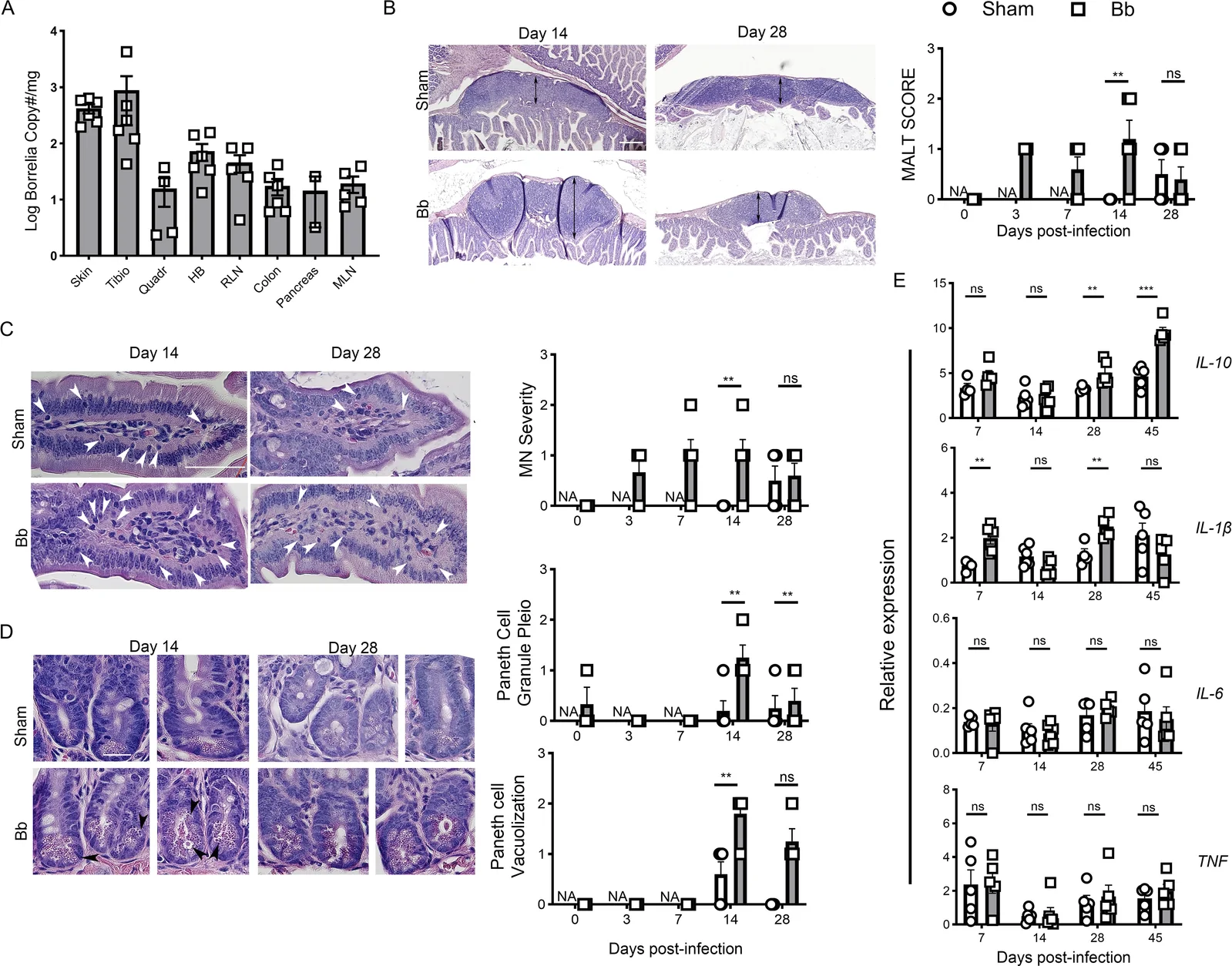
**Bb colonization and early inflammatory responses in gut tissue of Bb-infected mice: C57BL/6 female mice were infected with Bb cN40 or sham-infected. (A)** Bb copy numbers in skin, tibiotarsal joints (Tibio), quadriceps muscle (Quadr), heart base (HB), right inguinal lymph nodes (RLN), colon, pancreas, and mesenteric lymph nodes (mLN), as measured by Bb flagellin B PCR at 30 dpi. **(B–D)** Pathological evaluation of small intestines. Histological images were acquired at 40× magnification except the MALT, which were acquired at 4× magnification. The scale bar indicates 50 μm. **(B)** Left, MALT height (double-headed arrow); right, summary of results. **(C)** Left, mononuclear (MN) cell infiltration (arrowheads); right, summary of results. **(D)** Left, eosinophilic Paneth cell vacuolization and granulate size pleiomorphism (arrowheads); right, summary of results. **(E)** Relative cytokine mRNA expression in small intestines at indicated dpi, assessed by qRT-PCR. Statistical analyses were conducted using the Mann–Whitney U test. Bars indicate the mean ± SEM; each symbol represents one mouse (*n* = 3–6 per group). Each time point represents one independent experiment. **P ≤ 0.01, ***P ≤ 0.001, and ns, not significant (P > 0.05); NA, not available.

**Figure S1.**
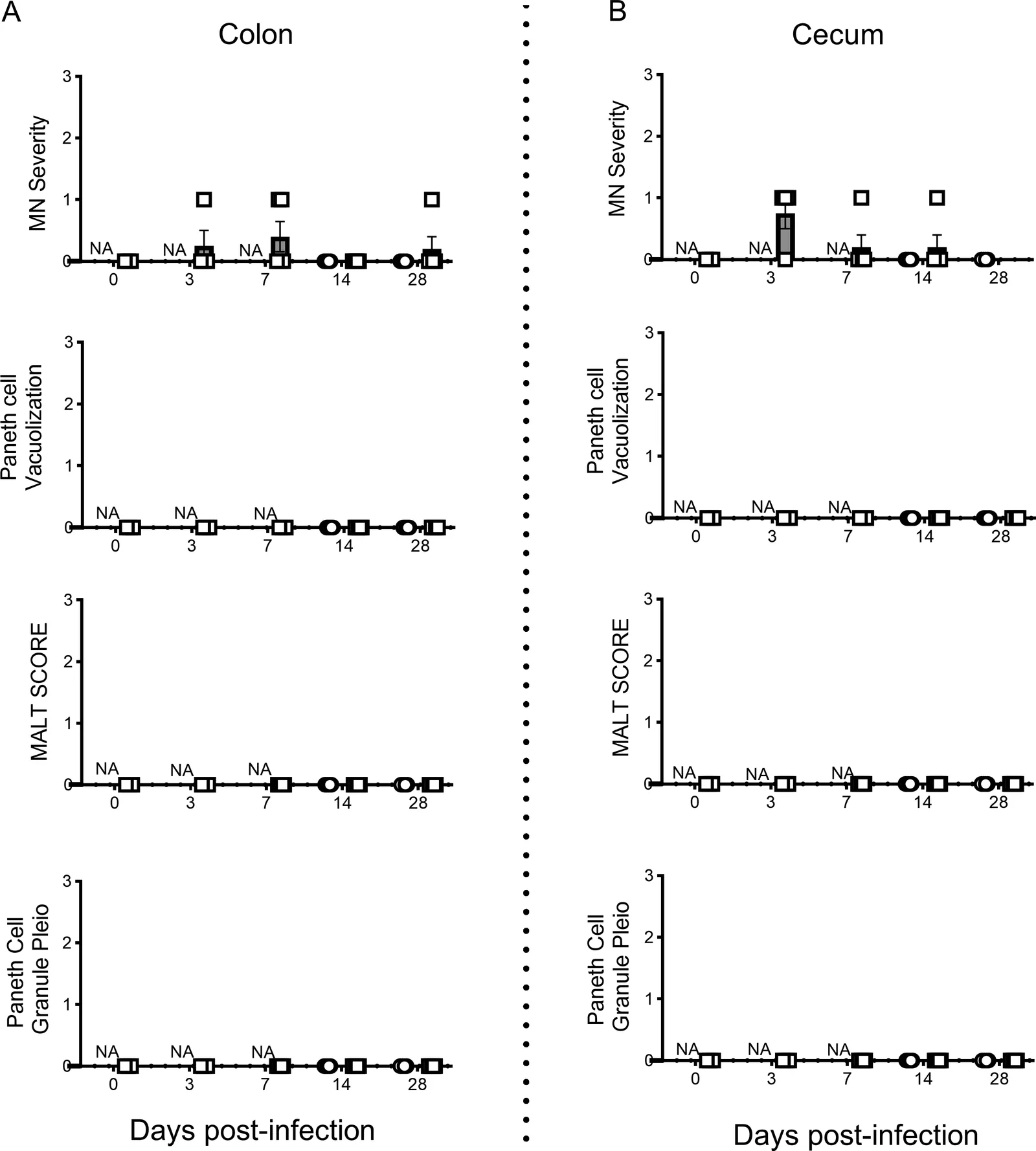
**Pathological evaluation of colon and cecum during Bb infection. (A and B)** Summary of the results. Statistical analyses were conducted by the Mann–Whitney U test; ns, not significant (P > 0.05).

Assessment of Bb presence in the GI tract of C57BL/6 mice 30 days postinfection (dpi) with cN40 Bb demonstrated their presence in all host tissues tested, including the colon, pancreas, and mesenteric lymph nodes (Fig. 1 A). Analysis was done via hematoxylin and eosin staining of GI tissues applying a histological scoring system devised for this purpose (Table S1). Histological analysis of the cecum and colon did not show evidence of inflammation (Fig. S1). Consequently, our subsequent analyses focused on the small intestine, where a significant enlargement of mucosal-associated lymph tissues (MALTs) in mice at 14 dpi compared with sham-infected controls was observed (Fig. 1 B), indicating an active local immune response to infection. Capillaries of the intestinal villi contained mononuclear and red blood cells, and mild increases in mononuclear cell infiltration were noted in surrounding tissues (Fig. 1 C). The intestinal crypts revealed mild-to-moderate increases in Paneth cell vacuolization at 14 and 28 dpi (Fig. 1 D), characterized by clear cytoplasmic spaces and granule size pleiomorphism, indicating variability in their secretory granules during the acute phase of infection (Fig. 1 D).

Measurement of key cytokines supported the presence of Bb infection–induced mild inflammation in the small intestine with significant upregulation of IL-1β from 7 to 28 dpi, but not IL-6 nor TNF-α. IL-10 significantly increased starting after 14 dpi with increases persisting until at least 45 dpi (Fig. 1 E). Thus, Bb infection induces local immune activation within the intestinal mucosa, indicating a functional impact of infection on mucosal epithelial defenses.

### Bb infection leads to gut leakiness and endotoxemia

**Figure 2.**
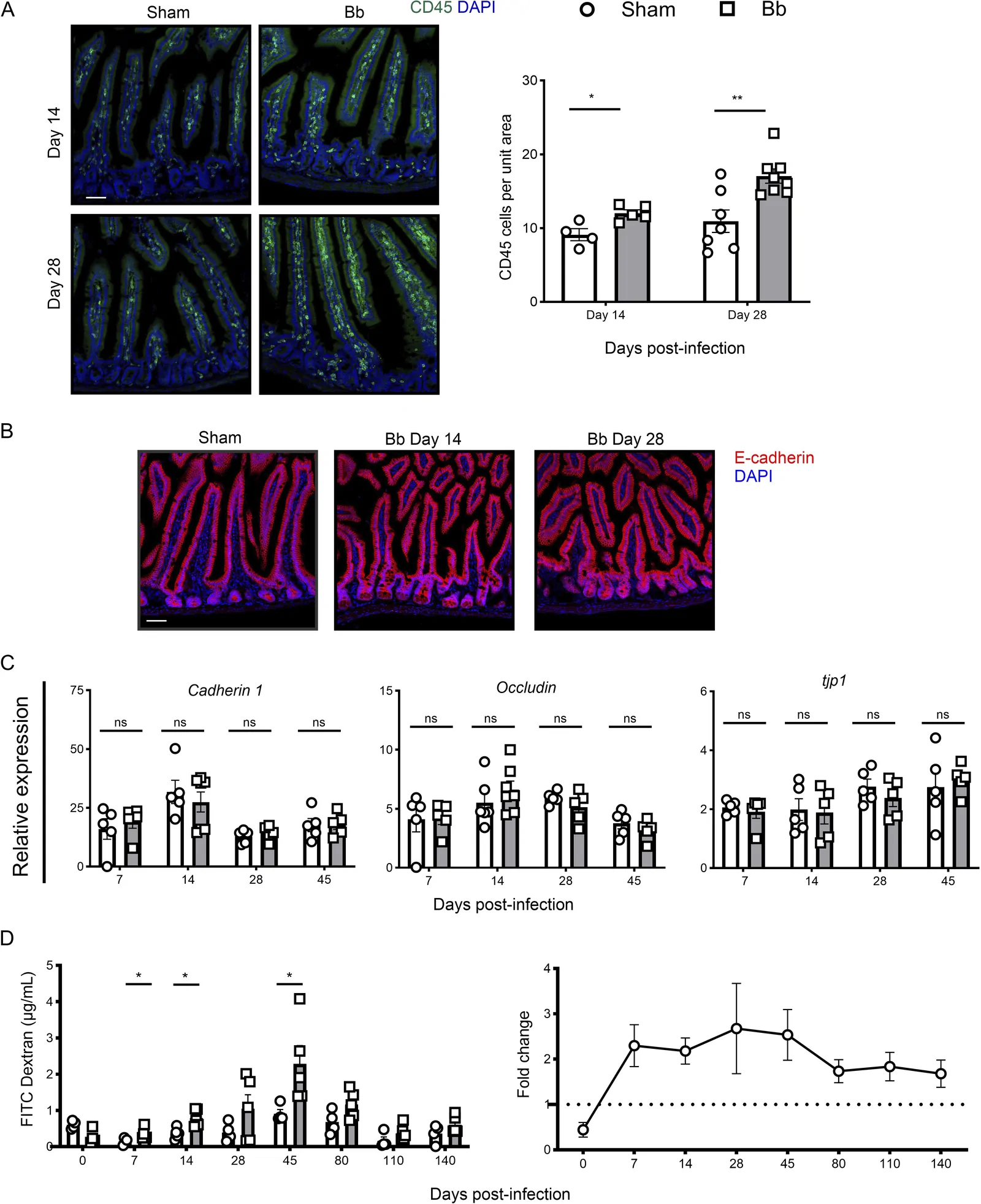
**Bb induces GI tract permeability without obvious alterations to its structural integrity.** All images were acquired at 20× magnification. The scale bar indicates 50 μm. **(A)** Left, fluorescent images of the small intestine from sham and Bb-infected mice at 14 and 28 dpi stained for CD45 (green) and DAPI (blue); right, summary of results. **(B)** Fluorescence staining of E-cadherin (red) and DAPI (blue) in the small intestine of sham- and Bb-infected mice at 14 and 28 dpi. **(C)** Relative mRNA expression of tight junction markers cadherin-1 and occludin, and tjp1 in the small intestine of sham and Bb-infected mice at the indicated dpi. **(D)** Left, serum FITC-dextran concentrations in serum of Bb- and sham-infected mice 4 h after intragastric administration of FITC-dextran at indicated dpi; right, data are shown as fold change in Bb-infected mice compared with the mean of the sham group at each time point. Statistical analyses were conducted using the Mann–Whitney U test. Bars indicate the mean ± SEM; each symbol represents data from one mouse (*n* = 4–8 per group). Each time point represents one independent experiment (A–C) except for days 28 in A and 14 in C, which was done twice. (D) Longitudinal data represent the same group followed over time with results on day 28 conducted in at least two additional independent studies that showed statistical significant differences between sham- and Bb-infected mice. *P < 0.05, **P ≤ 0.01, and ns, not significant (P > 0.05). tpj1, tight junction protein 1.

**Figure S2.**
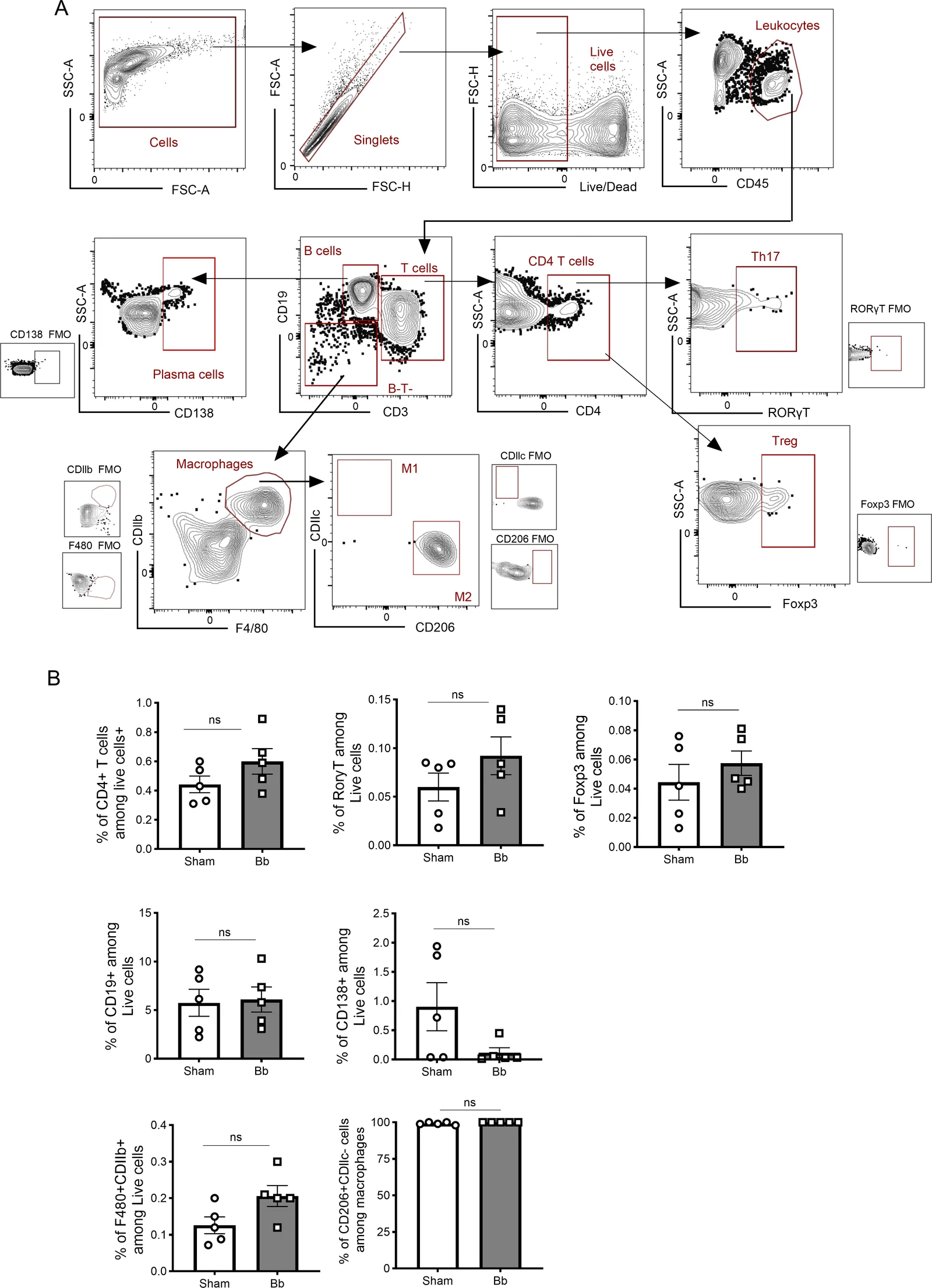
**Phenotypic characterization of lamina propria immune cell populations following Bb infection. (A)** Representative flow cytometric contour plots with outliers illustrating the gating for Th17 cells, Tregs, plasma cells, and M1/M2 macrophages. **(B)** Summary of the results. Bars indicate the mean ± SEM, and each symbol represents an individual mouse (*n* = 5 per group). Data are representative of pooled data from two independent experiments. Statistical analyses were conducted by the Mann–Whitney U test. ns, not significant (P > 0.05).

Consistent with the observed histological and cytokine expression changes, immunofluorescence imaging revealed significant increases in leukocyte numbers (CD45^+^) within the small intestine of Bb-infected mice at both 14 and 28 dpi compared with the sham group (Fig. 2 A). Immunophenotyping of lamina propria T cells, B cells, and macrophage subsets by flow cytometry at 14 dpi showed modest differences in major cell populations. No changes in B cell or T cell frequencies were observed, including frequencies of RORγt^+^ Th17 or FoxP3^+^ regulatory T cells (Tregs) compared with sham-infected mice. There were significant reductions in plasma cell frequencies among Bb-infected mice, as well as increases in total macrophage populations. Macrophages skewed toward a CD206^+^ CD11c^lo^ M2-like phenotype, suggesting a lack of robust pro-inflammatory polarization (Fig. S2).

**Figure S3.**
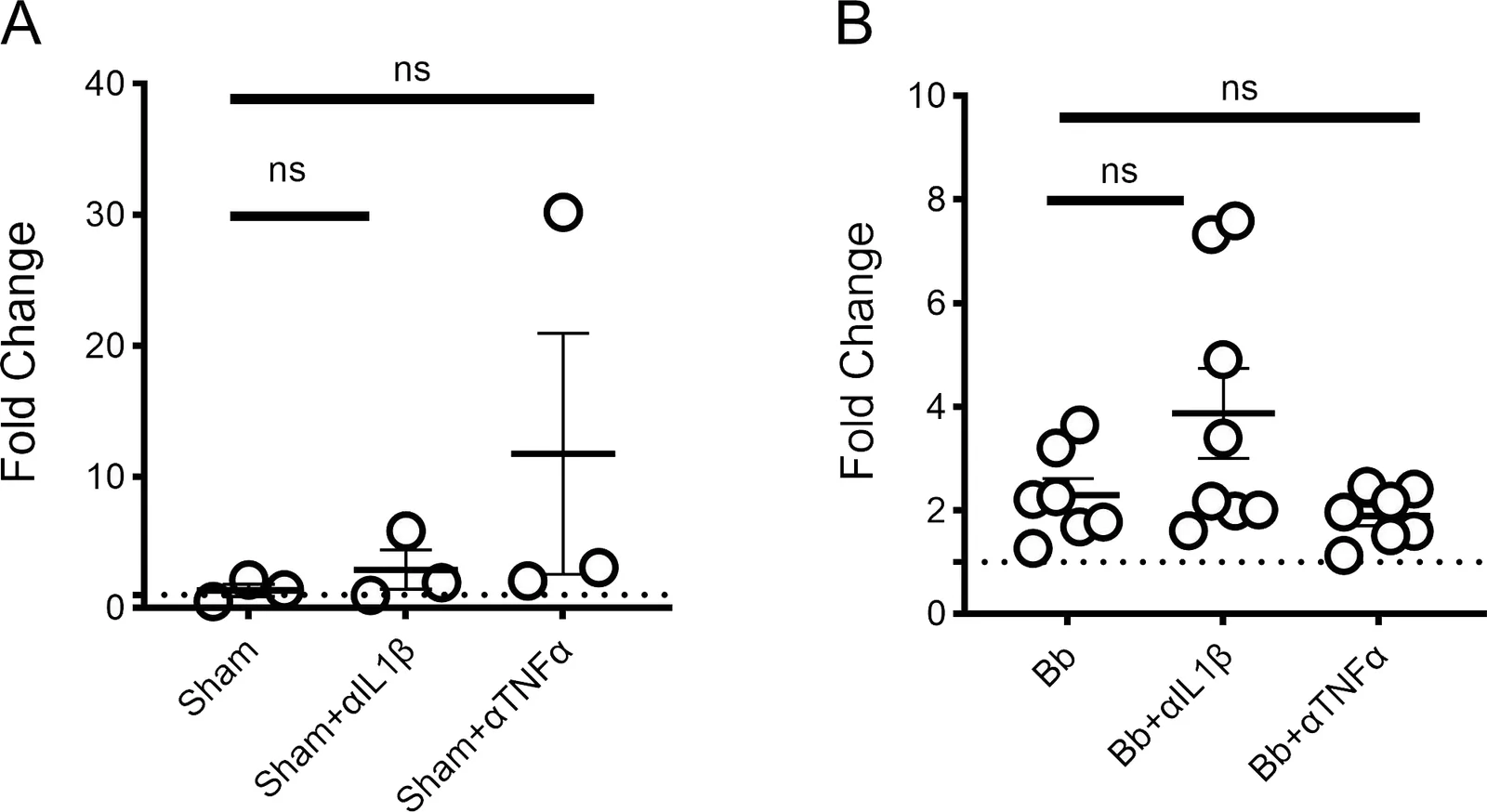
**Effect of IL-1β and TNF-α neutralization on intestinal permeability during Bb infection. (A and B)** Data represent the fold change in serum FITC-dextran concentrations 4 h after gastric application at day 14 after infection compared with day 0 in (A) sham-infected and (B) Bb-infected mice treated with mAb to IL-1β or TNF-α. Bars indicate the mean ± SEM, and each symbol represents an individual mouse (*n* = 3/group in A; *n* = 8/group in B). Results in A are from one experiment; B shows pooled data from two independent experiments. Statistical analyses were done by one-way ANOVA; ns, not significant (P > 0.05).

The structural integrity of the intestinal barrier appeared unaltered, with E-cadherin staining showing no obvious gross structural alterations between Bb-infected and sham-infected mice with the exception of what appears to be a shortening of intestinal villi (Fig. 2 B). mRNA expression levels of key tight junction proteins were also unaltered (Fig. 2 C). Functional alterations were, however, observed with significant increases in GI tract permeability measurable as early as 7 dpi for the duration of infection (140 dpi). This was assessed by measuring the translocation of FITC-dextran, given to mice via esophageal gavage, across the gut mucosa into the serum (Fig. 2 D). Neither the Bb-induced increased local production of IL-1β (Fig. 1 E) nor the presence of TNF-α affected the increased gut permeability of Bb-infected mice, as blocking these cytokines via mAb treatment did not reduce the enhanced FITC-dextran translocation at 14 dpi seen after Bb infection compared with controls (Fig. S3). Collectively, these findings show that Bb infection induces mild inflammation of the small GI barrier with some structural alterations and functionally enhanced gut permeability. The mechanisms inducing this early functional defect induced by Bb infection remain to be further evaluated but are unrelated to the enhanced induction of local IL-1β production.

**Figure 3.**
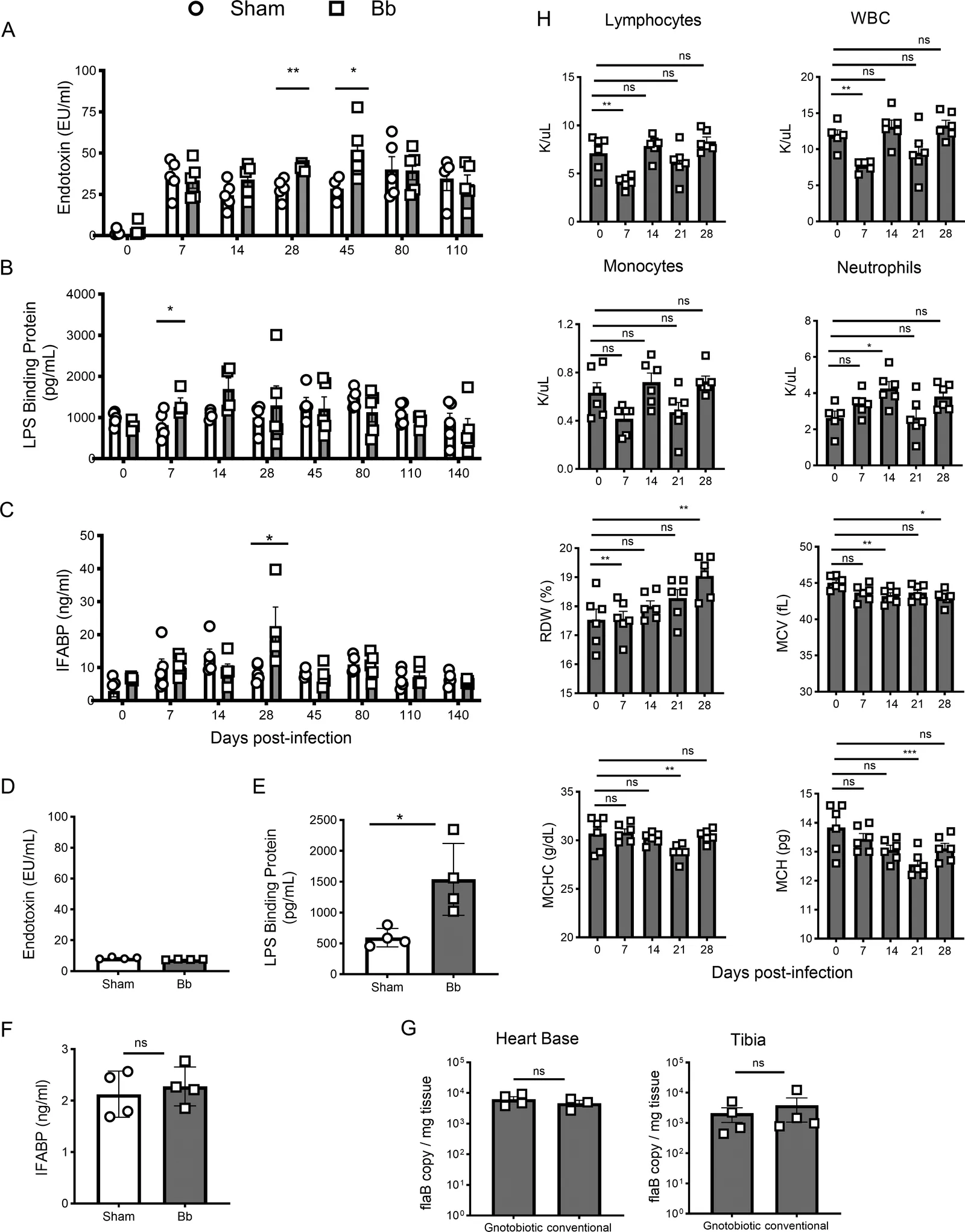
**Bb infection causes changes to white and red blood cell counts and hallmarks of leaky gut syndrome. (A–C)** ELISA analysis of serum samples from Bb-infected and age-matched sham controls at indicated dpi for (A) endotoxin, (B) LBP, and (C) IFABP. **(D–G)** Gnotobiotic Swiss Webster mice infected intradermally with Bb or sham-infected. Serum levels (E), endotoxin, (F) LBP, and (F) IFABP at 21 dpi. **(G)** Bb load in germ-free and conventional SPF-held mice at 21 dpi. **(H)** CBC of Bb-infected C57BL/6 mice at indicated dpi. Upper graphs display absolute leukocyte counts of indicated cell types; lower graphs summarize red cell assessment, including red cell distribution width (RDW), mean corpuscular volume (MCV), mean corpuscular hemoglobin concentration (MCHC), and mean corpuscular hemoglobin (MCH). Bars represent the mean ± SEM, with each symbol corresponding to one mouse. Longitudinal data (A–C and H) are from one group of mice (*n* = 6) followed over time. Statistical analyses were conducted using the Mann–Whitney U test. *P < 0.05, **P ≤ 0.01, ***P ≤ 0.001, and ns, not significant (P > 0.05).

The increases in gut permeability following Bb infection correlated with transient increases in serum LPS levels at 28 and 45 dpi (Fig. 3 A). Consistent with the induction of “gut leakiness” and systemic inflammation, serum levels of LPS binding protein (LBP) were significantly upregulated between 7 and 14 dpi (Fig. 3 B), as were intestinal fatty acid binding protein (IFABP) levels at 28 dpi (Fig. 3 C). Bb does not express classical LPS, and its presence cannot explain the observed endotoxemia. Because germ-free–held, intradermally infected Swiss Webster mice failed to develop endotoxemia or changes in serum IFABP levels, these findings indicate that the changes observed in the specific pathogen–free (SPF)-held mice were due to microbiome translocation. LBP on the other hand showed strong induction compared with sham-infected mice, indicating this as a marker of Bb infection rather than a secondary effect of microbiome translocation (Fig. 3, D–F). Both gnotobiotic and SPF-held mice had similar levels of Bb tissue burden following intradermal infections (Fig. 3 G). In addition to markers of gut barrier dysfunction, the broader systemic inflammatory environment in Bb-infected mice was characterized by assessment of complete blood counts (CBCs), demonstrating significant reductions in total white blood cells (WBCs) and lymphocytes during the acute early phase of infection, as well as significantly elevated neutrophil counts by 28 dpi (Fig. 3 H). Monocyte counts remained unchanged throughout the observed period. Erythrocyte parameters were also significantly altered at later stages of infection, including red cell distribution width, mean corpuscular volume, mean corpuscular hemoglobin concentration, and mean corpuscular hemoglobin, most prominently at 21 and 28 dpi (Fig. 3 H). Collectively, these results demonstrate that Bb infection induces gut barrier dysfunction, resulting in a systemic leaky gut syndrome, likely triggered by host inflammatory responses to Bb infiltration of GI tract mucosal tissues following their dissemination from the skin after infection.

### Lyme disease patients show evidence of systemic inflammation

**Figure 4.**
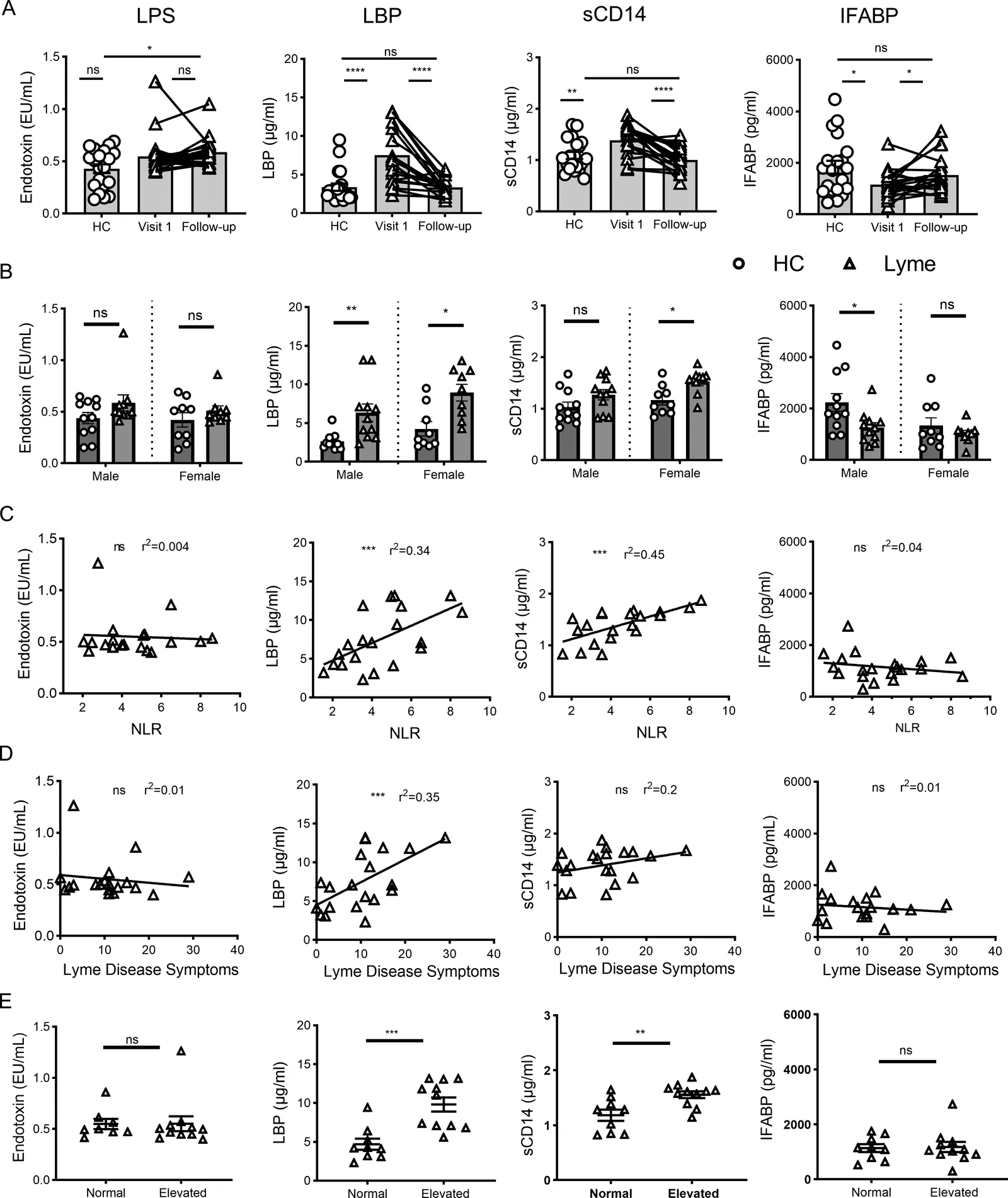
**Lyme disease patients show hallmarks of systemic inflammation. (A)** ELISA analysis of serum concentrations of endotoxin, LBP, sCD14, and IFABP in 20 Lyme patients (visit 1 and 6-month follow-up after antibiotic treatment) compared with 20 age-matched HCs. **(B)** Sex-based comparison of above serum markers between Lyme patients and age-matched HCs. **(C and D)** Correlation analysis between the above serum markers and the NLR (C), as well as with the number of acute-onset Lyme disease symptoms (D), in the Lyme patient group. **(E)** Comparison of the above serum marker levels in Lyme patients with normal liver function versus those with abnormal elevations in at least one of the following liver markers at visit 1: aspartate aminotransferase, alanine aminotransferase, or alkaline phosphatase. Bars indicate the mean ± SEM; each symbol represents results from single participant. (A) Mann–Whitney U test between HCs and V1 or follow-up, paired Student’s *t* test between V1 and follow-up. (B and E) Mann–Whitney U test. (C and D) Simple linear regression analyses.*P < 0.05, **P ≤ 0.01, ***P ≤ 0.001, ****P ≤ 0.0001, and ns, not significant (P > 0.05) ns, not significant (P > 0.05). sCD14, secretory CD14.

We previously showed that Lyme disease patients showed similar alterations to their CBCs prior to treatment onset (Rebman et al., 2024), especially changes to the neutrophil–lymphocyte ratio (NLR), which correlates with early Lyme disease severity (Rebman et al., 2024). Analysis conducted here using male and female Lyme disease patients (*n* = 20), most of whom presented to the clinic with multiple erythema migrans, a sign of disseminated Lyme disease (Table S2), showed significant increases in serum concentrations of LBP and sCD14 at visit 1 (antibiotic-naïve) compared with HCs. These increases had resolved by 6 months and after completing antibiotic treatment (Fig. 4 A). Levels of serum endotoxin were not significantly increased in the blood of Lyme disease patients at visit 1 compared with the HC group, and were not changed over time when comparing pre- and posttreatment samples (Fig. 4 A). In contrast to the increases in IFABP seen at 28 dpi in mice (Fig. 3 C), IFABP levels at visit 1 were significantly reduced in acutely infected patients compared with HCs and returned to normal levels by 6 months and antibiotic treatment (Fig. 4 A). Given the highly temporal changes in blood values observed in mice (Fig. 3), analysis at different time points during the infection may have resulted in different findings. Nonetheless, changes to IFABP are a feature of Lyme disease in humans that is responsive to antibiotic treatment. No gender differences were noted for any measured changes (Fig. 4 B), although some of the differences reached statistical significance for only one gender, likely due to the smaller numbers of patients per group.

**Figure S4.**
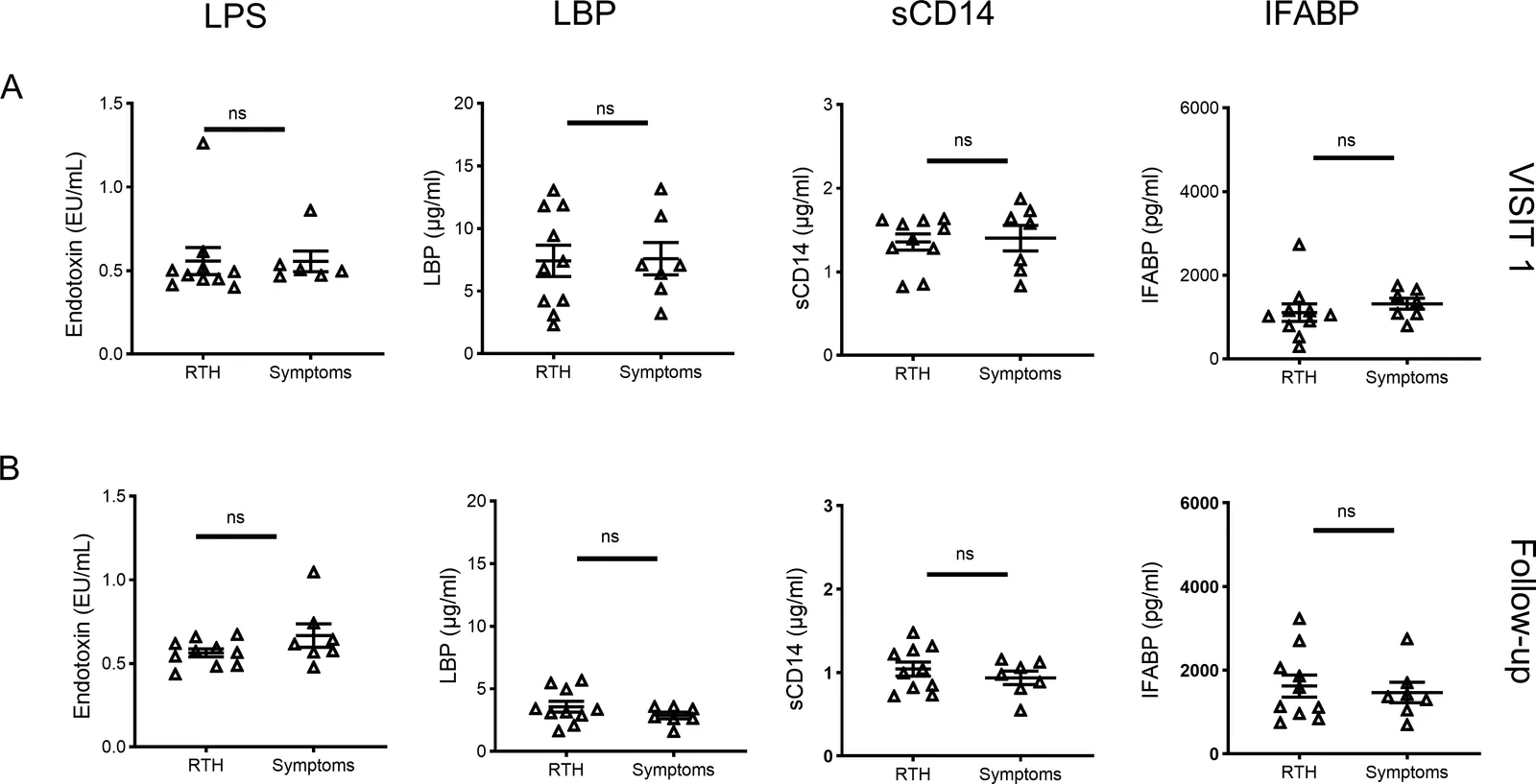
**Serum biomarkers in Lyme disease patients returning to health versus those with long-term symptoms. (A and B)** Comparison of the indicated serum markers at visit 1 (A) and follow-up visit (B), between individuals who returned to health (RTH) and those who remained symptomatic (symptoms). Bars indicate the mean ± SEM; each symbol represents results from single participant. Statistical analyses were conducted using the Mann–Whitney U test; ns, not significant (P > 0.05).

LBP and sCD14 serum levels significantly correlated with the NLR, underscoring the systemic effects of Bb infection on the immune system (Fig. 4 C). Strong correlations existed also between LBP and sCD14 levels and the number of Lyme disease symptoms experienced in patients at disease presentation, consistent with prior reports of a correlation between increased NLR and disease severity (Fig. 4 D and Table S2; Rebman et al., 2024). Of note, LBP and sCD14 levels were significantly higher in individuals presenting with transient altered liver function measurements during acute infection compared to those with normal liver function (Fig. 4 E). Finally, analysis of Lyme disease patients who returned to health (*N* = 10) compared with those who showed continued (>6 months) symptoms (*N* = 7) showed no measurement differences at either visit 1 (Fig. S4 A) or follow-up (Fig. S4 B). However, a caveat for this analysis is that the study was not sufficiently powered to distinguish measurements between disease outcomes and larger patient cohorts would have to be obtained for robust conclusions.

Thus, acutely infected Lyme disease patients showed significant changes to their WBCs, LBP, and sCD14 that were correlated with disease severity at presentation and mostly resolved with antibiotic treatment. In contrast to mice that showed significant endotoxemia starting around day 28, the small increases in endotoxin levels in the blood noted in the Lyme patient group compared with HCs did not resolve with antibiotic treatment. Whether this difference is due to timing of analysis since infection/symptom appearance (Table S2) is difficult to ascertain, as the exact lengths of infection of these patients are unknown.

### Bb infection impairs B cell immunity in the GI tract

**Figure 5.**
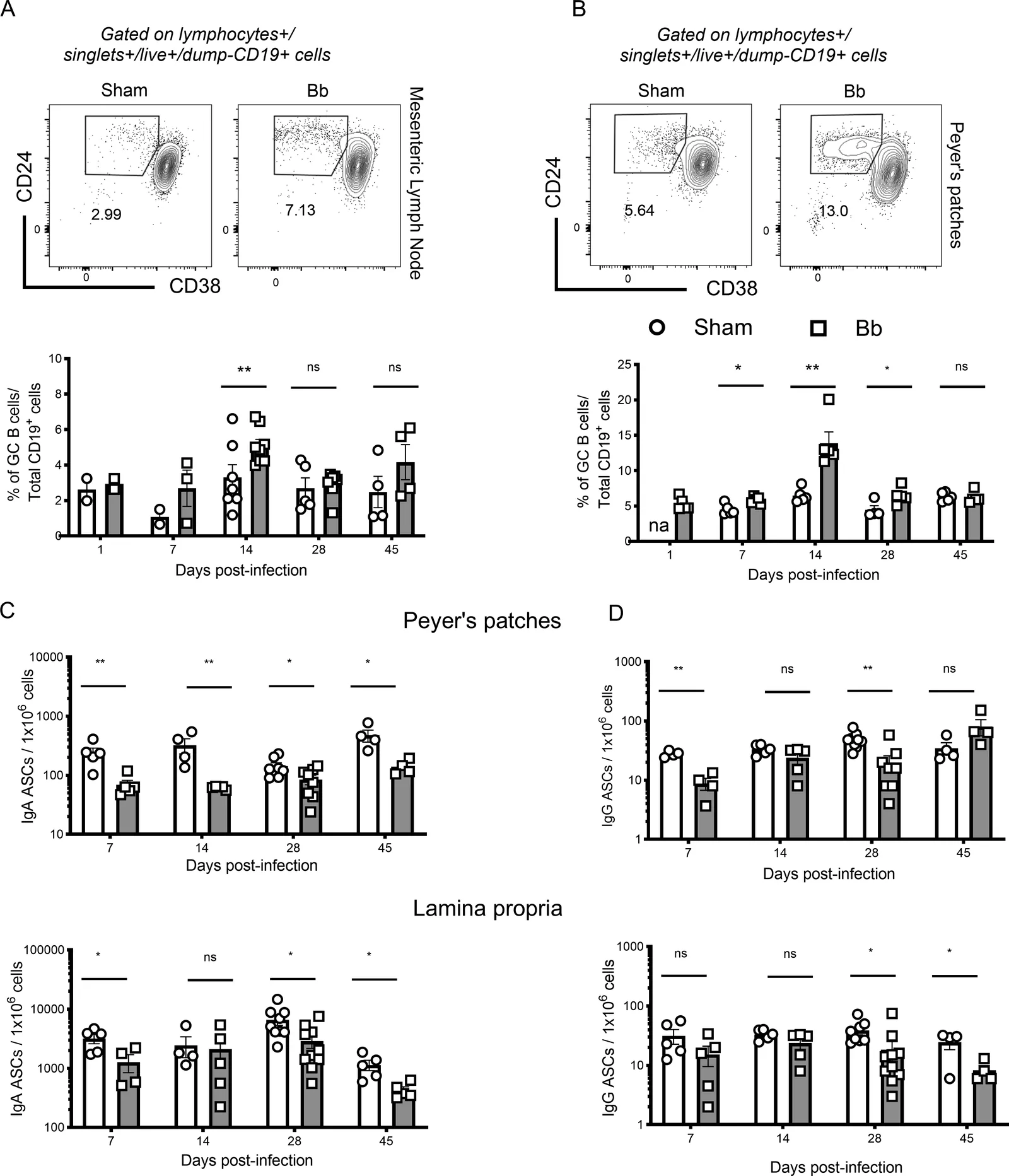
**Bb alters local B cell responses in the GI tract. (A and B)** Top, representative flow cytometry contour plots of GC B cells, defined as CD24^hi^ CD38^low^ cells gated on CD19^+^ live singlet lymphocytes, in (A) mesenteric lymph node and (B) Peyer’s patches at 14 dpi with Bb- or sham-infected controls. Lower panels, quantification of GC B cells as the percentage of total B cells. **(C)** Quantification of IgA ASCs in Peyer’s patches and lamina propria at indicated dpi, as measured by the ELISpot assay. **(D)** Quantification of IgG ASCs in Peyer’s patches and the lamina propria at indicated dpi, assessed by ELISpot. Bars indicate the mean ± SEM; each symbol represents results from one mouse (*n* = 2–10 per group). Each time point represents an independent experiment (day 14: A and B; day 28: C and D), each performed twice. Statistical analyses were conducted using the Mann–Whitney U test; *P < 0.05, **P ≤ 0.01, and ns, not significant (P > 0.05); NA, data not available.

Given the crucial role of humoral immunity in maintaining a functioning mucosal barrier and the suppression of systemic B cell responses observed during Bb infection, we next assessed B cell responses in the GI tract of Bb-infected mice. As expected, mesenteric lymph nodes and Peyer’s patches harbored GC B cells in the absence and presence of Bb infection at all time points assessed. In contrast to the sham-infected group, Bb-infected mice showed transient increases in GC populations around day 14 of infection, which returned to noninfection levels by 28 dpi (Fig. 5, A and B), a time point at which we previously observed systemic contraction of GC responses (Elsner et al., 2015; Hastey et al., 2012).

Secretory IgA is a primary defense mechanism of mucosal barriers. It can neutralize pathogens and maintain gut homeostasis by binding to and inhibiting the luminal microbiota to invade the host (Pabst and Slack, 2020). B cell ELISpot analysis of Peyer’s patches and intestinal lamina propria cells revealed significant reductions in relative and absolute numbers of IgA antibody–secreting cells (ASCs) in Bb-infected mice compared with sham controls across several time points, starting as early as 7 dpi with Bb (Fig. 5 C). Similar reductions were noted in local IgG-ASC frequencies (Fig. 5 D). Although IgG levels in the GI tract are low, IgG was shown nonetheless to contribute to mucosal protection against pathogens that may penetrate the epithelial barrier, or mediate inflammatory responses (Eckmann and Stappenbeck, 2015; Pickard et al., 2017). Thus, Bb infection suppresses local antibody responses in the GI tract, much of which is thought to be directed against microbiota residing in the gut lumen.

### The microbiota of Bb-infected mice changes over time

**Figure 6.**
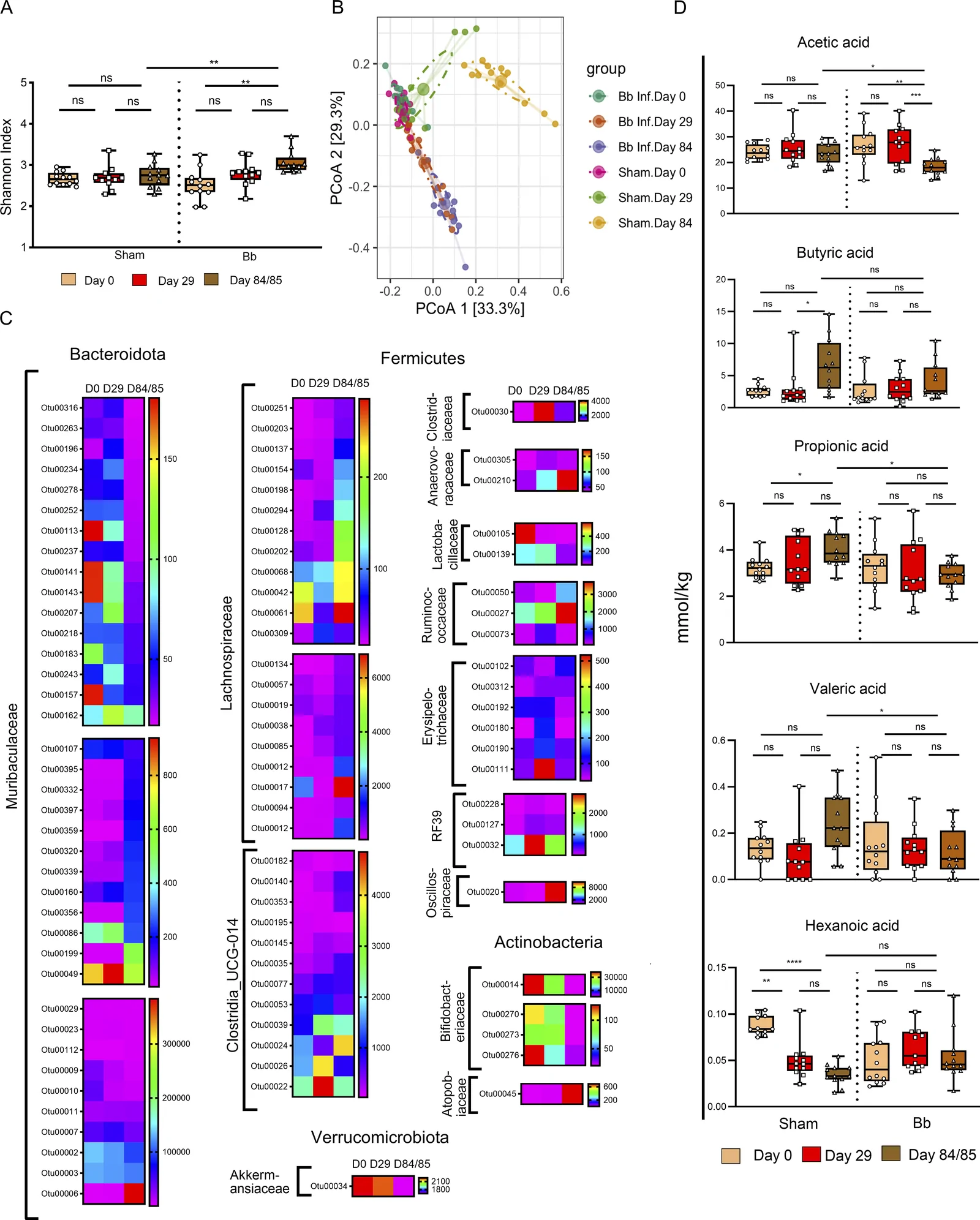
**Changes to the GI microbiome of Bb-infected mice.** Fecal samples were collected from Bb-infected and control sham-infected mice (*n* = 12/group) at 0, 29, and 84/85 dpi for 16S rRNA sequencing and SCFA analyses. **(A)** Mean ± SEM α diversity of fecal microbiota (Shannon index). **(B)** PCoA of β diversity, assessed by calculating Bray–Curtis dissimilarities from OTU abundance. **(C)** Heat map representing the mean read counts of OTUs (Data S1) that showed significant changes in either or both day 29 and day 84/85 Bb mice compared with sham-infected controls. **(D)** Mean ± SEM concentrations of indicated SCFAs. Each symbol represents data from one mouse. **(A–D)** Each time point represents part of a longitudinal study following the same group of mice over time. One-way ANOVA, *P < 0.05, **P ≤ 0.01, ***P ≤ 0.001, ****P ≤ 0.0001, and ns, not significant (P > 0.05). PCoA, principal coordinate analysis.

To assess the impact of Bb infection and the ensuing “leaky” gut on microbial communities in the lumen of the GI tract, 16S rRNA sequencing was performed on longitudinally collected fecal samples of mice infected with Bb or sham-infected at 0, 29, and 84/85 dpi. Analysis of α diversity revealed significant alterations in gut microbial community richness and evenness in the Bb-infected but not the sham-infected group over time (Fig. 6 A). Principal component analysis of the microbial community profiles, providing a visual representation of the overall similarities and differences between groups and time points, supported these findings by demonstrating a clear separation and distinct clustering of the Bb-infected groups at 29 and 84/85 dpi compared with the samples taken prior to infection. The samples showed also clear differences compared with the sham-infected samples at all time points, which clustered together (Fig. 6 B).

Significant changes in read abundance, normalized to the sham-infected control group, indicate dynamic alterations in the gut microbiota composition of Bb-infected mice over the course of infection, with notable operational taxonomic unit (OTU)-level shifts in Bacteroidota, Firmicutes, Actinobacteriota, and Verrucomicrobiota (Fig. 6 C and Data S1). Consistent with Bb-induced microbial alterations, significant shifts in the most abundant short-chain fatty acids (SCFAs), acetic acid, were found in Bb-infected but not sham-infected mice (Fig. 6 D). Significant increases in propionic acid and decreases in hexanoic acid occurred in sham but not Bb-infected mice. Together, these data suggest functional metabolic shifts in the gut microbiome following Bb infection (Fig. 6 D).

**Figure S5.**
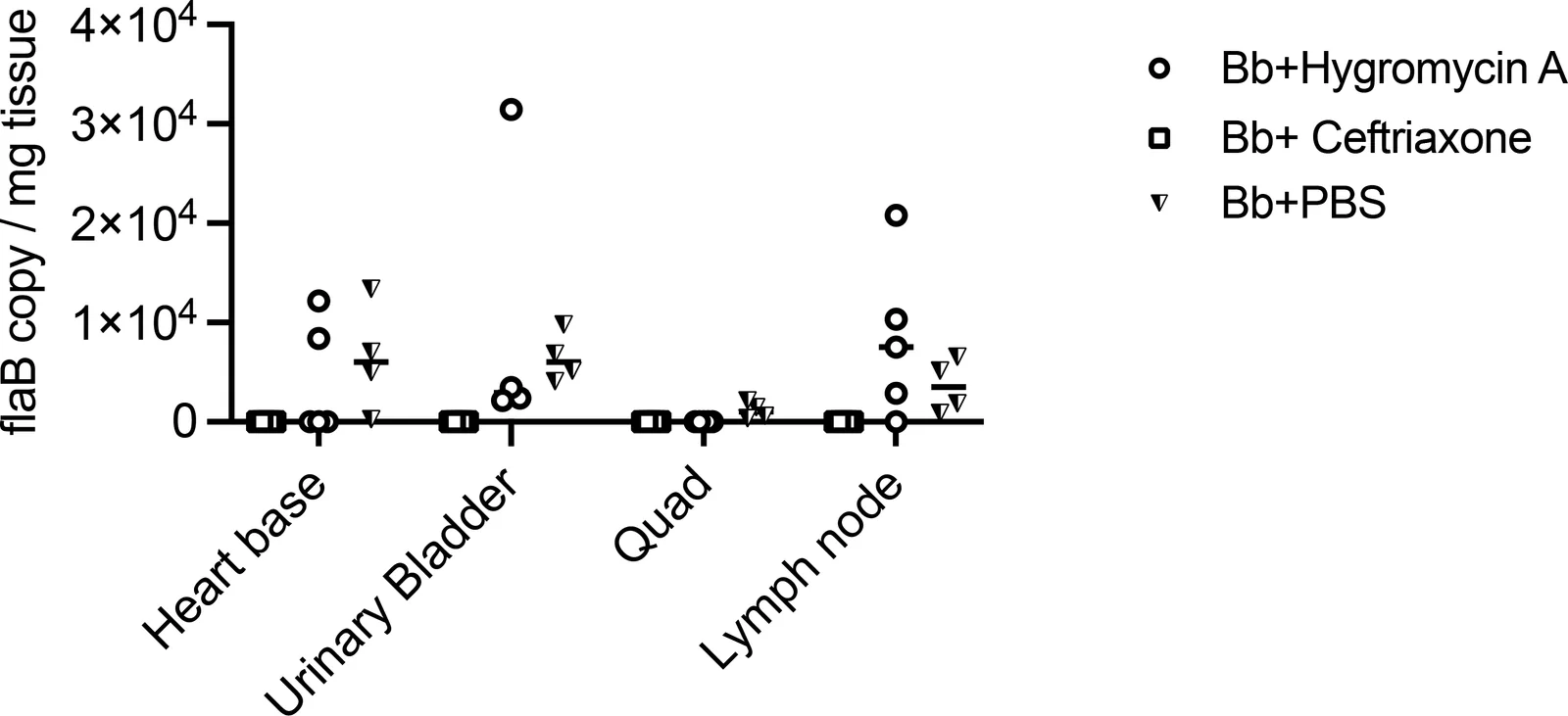
**Analyses of Bb tissue burden following antibiotic treatment.** Bb copy numbers (*n* = 3–5/group) in indicated tissues at 210 dpi from mice receiving a course of treatment with ceftriaxone or hygromycin A, or who were sham-treated. All treatments were initiated at 45 dpi; Quad, right quadriceps. Data represent results from one study of two conducted that gave similar results.

We aimed to assess how the selective removal of Bb spirochetes would affect the microbial communities of the GI tract, treating a group of mice with the hygromycin A as this antibiotic was reported to remove selectively Bb in mice when analyzed 24 h after the end of treatment, but to have no or only little effect on other microbial communities (Leimer et al., 2021), and comparing it with a sham-treated group of mice and a third group of mice treated with ceftriaxone, which we and others have shown to clear Bb from infected hosts, but which is expected to have significant effects on the microbiota (Elsner et al., 2015; Moody et al., 1994). Unexpectedly, in two independent experiments using hygromycin A from two different sources, and following treatment strategies previously reported to deplete Bb in mice but using twice the previously used dose, PCR analysis detected similar levels of Bb DNA in tissues from mice treated with either hygromycin A or PBS, when analyzed 5 months after the end of the treatment. In contrast, and as expected, Bb was not detectable in mice treated with ceftriaxone and analyzed at the same time as the other two groups (Fig. S5). Given the expected broad effects of ceftriaxone on microbial communities, we did not assess the microbiome of the treated animals. We conclude that hygromycin A is unable to fully clear Bb infection in mice, precluding us from distinguishing between primary effects of Bb on the microbial communities versus those induced indirectly through the suppression of IgA production.

### Reduced ex vivo IgA coating of luminal microbiota in Bb-infected mice

**Figure 7.**
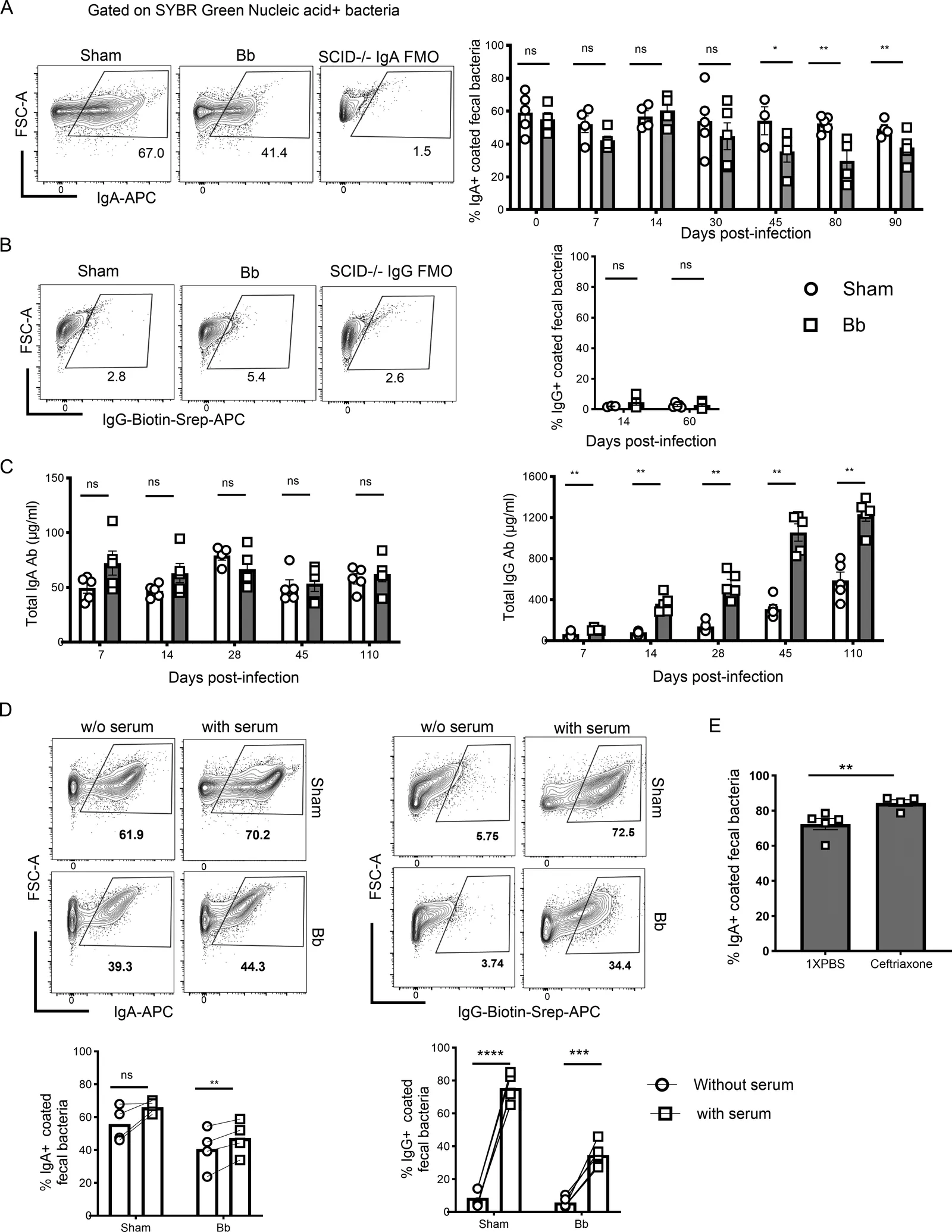
**Loss of IgA binding to gut microbiota after Bb infection. (A and B)** Left, representative FACS contour plots with outliers showing (A) IgA and (B) IgG staining of SYBR Green nucleic acid–positive microbiota isolated from the feces of Bb-infected and sham-infected mice at 45 dpi; right panel, summary of results. **(C)** Total (left) IgA and (right) IgG in the serum of Bb- and sham-infected mice at indicated dpi. **(D)** Upper panel represents FACS contour plots of fecal microbiota isolated from of Bb-infected and sham-infected mice at 90 dpi and incubated with and without serum for detection of IgA (left) and IgG (right) binding. Lower panel, summary of results. **(E)** Mean ± SEM frequencies of IgA-coated bacteria in feces of Bb-infected mice collected at 90 dpi and 15 days after the end of 30-day treatment with ceftriaxone or sham treatment with PBS. Each symbol represents results from one mouse (*n* = 4–5/group). **(A–C)** Each time point represents one longitudinal analysis of samples from the same mice. D and E represent one independent experiment. Mann–Whitney U test (A–C and E); paired Student’s *t* test (D); *P < 0.05, **P ≤ 0.01, ***P ≤ 0.001, ****P ≤ 0.0001, and ns, not significant (P > 0.05).

Bb FlaB DNA was not detected in fecal matter, suggesting that the reduction in IgA and/or IgG ASCs within GI tissues is a primary driver of Bb infection–induced changes to the composition of the microbiota, allowing the immunological escape and translocation of bacteria from the gut lumen into the bloodstream. To assess this, we stained bacteria enriched from fecal pellets of both Bb-infected and sham-infected mice for surface-bound IgA and IgG, by flow cytometry, using the nucleic acid dye SYBR Green to identify DNA-containing bacteria. As expected, the majority of bacteria in sham-infected or noninfected C57BL/6 mice were coated with IgA, while those from B cell–deficient SCID mice showed little appreciable IgA binding (Fig. 7 A). Starting from 30 dpi with Bb, IgA coating of the retrieved microbiota was significantly reduced compared with the sham-infected mice over the 90 days of analysis (Fig. 7 A). Only relatively small amounts of IgG-coated bacteria were detected in either the sham or Bb-infected group (5.8% ± 3.11 and 8.6% ± 5.2, respectively; Fig. 7 B).

In contrast to local reductions in IgA production, Bb infection did not impact serum IgA levels (Fig. 7 C), but caused significant increases in serum IgG concentrations (Fig. 7 C). After incubation of serum IgA from either the sham-infected or the Bb-infected group with each mouse’s own microbiota, frequencies of IgA^+^ bacteria only marginally increased, with increases reaching significance among the Bb-infected mice (Fig. 7 D). The latter group had overall lower frequencies of IgA-coated microbiota both ex vivo and after incubation with serum than the sham-infected group. In contrast, serum IgG from both sham- and Bb-infected mice strongly bound to fecal-derived bacteria, increasing their frequencies (Fig. 7 D). Yet, despite the significantly higher total serum IgG concentrations in Bb-infected mice, serum IgG binding to microbiota conducted at the same serum dilution was only about half of that seen in the sham group (34.6% ± 4.5 versus 75.4% ± 8.4; P < 0.0001; Fig. 7 D), i.e., a sixfold increase compared with a nearly ninefold increase in the sham group.

We further examined whether antibiotic clearance of Bb affects IgA coating of the microbiota. For that, groups of mice were infected with Bb and treated at 45 dpi with either ceftriaxone for 30 days, or with PBS. 15 days after the end of treatment (90 dpi), ceftriaxone-treated mice showed significant increases in IgA-coated bacteria compared with the Bb-infected and sham-treated controls (Fig. 7 E).

Thus, Bb infection causes the inhibition of effective antimicrobiome humoral responses, including both local IgA and systemic IgG responses, a process that seems reversible with effective antibiotic treatment. These immune alterations seem to compromise gut barrier function and to drive changes to the GI microbiota.

## Discussion

The study demonstrates that Bb-infected mice develop a leaky gut syndrome. Given the kinetics of the observed changes in Bb-infected mice, we propose a scenario whereby Bb dissemination and invasion of mucosal tissues cause mild local and systemic inflammation, followed by suppression of local and systemic functional antibody responses, including to the microbiota, which results in increasing changes to the microbial communities and their metabolites in the GI tract over time. Our findings identify a key mechanism by which Bb infection can impact overall immune system health, beyond that caused by the pathogen itself. It also suggests that secondary changes to the microbiota might impact overall health long after antibiotic treatment successfully removes Bb. Acute Lyme disease patients exhibit changes to both cellular and soluble inflammatory markers that were associated with measures of Lyme disease severity, including alterations to liver function values, several correlated with the changes observed in infected mice. We conclude that Bb infection induces mucosal barrier dysfunction and secondary changes resulting from it.

Microbial translocation and subsequent endotoxemia are well described in other chronic inflammatory conditions, such as HIV infection, and critical illnesses like sepsis, where intestinal barrier dysfunction contributes significantly to systemic inflammation and disease progression (Deeks et al., 2013; van Lier et al., 2019). Mice showed transient endotoxemia around 28 dpi, while endotoxemia was not observed in Lyme disease patients, a finding that could be due to the time points at which samples were available from the human study. Consistent between mice and humans, the LBP was significantly elevated during acute infection and in the patient group returned to levels seen in HC patients by 6 months after treatment. Alterations in total WBC counts and changes to the NLR that had been associated previously with the severity of illness (Rebman et al., 2024) were also similar in mice and humans. Bb infection induced IFABP changes in both mice and humans, in the latter measured at visit 1. Antibiotic treatment of patients and measurement at the 6-month mark showed return to levels comparable to that of the HC group, suggesting that the changes depend on active Bb infection. However, while IFABP levels increased in mice at 28 dpi, in humans, IFABP levels were significantly decreased at visit 1. Similar to the apparent discrepancy seen with serum LPS levels, which were not elevated in humans, in contrast to mice, this discrepancy could be due to the timing of blood draws, given the highly time-dependent changes seen in mice, and pointing to the need for more frequent blood draws in infected humans. Whether all these shifts are a direct consequence of gut barrier dysfunction or a reflection of the broader systemic inflammatory burden across multiple Bb-infected tissues warrants further investigation.

Elevation of markers of acute innate inflammatory responses and liver enzyme changes in Bb-infected patient are consistent with a previous study reported on Bb infection–induced transient early increases in C-reactive protein and serum amyloid in human patients (Uhde et al., 2016). The study here provides an impetus for future studies that studies Lyme patients and their disease outcomes with measurement of changes to the GI tract. Treatment delay has previously been associated with an increased risk for Lyme patients to develop PTLDS. An ongoing infection or an increased systemic distribution of Bb in such patients may increase the risk of GI barrier dysfunction and downstream effects such as dysbiosis that may not necessarily be treated (or perhaps even be aggravated) by broad-spectrum antibiotic treatment (Bobe et al., 2021).

A central finding of our study is the substantial impairment of B cell immunity within the GI tract following Bb infection. These findings demonstrate that the previously observed suppression of humoral immunity against de novo antigens in Bb-infected mice (Elsner et al., 2015; Hastey et al., 2012) extents to diminishing established immune responses already present at the time of infection, with reductions notable in both intestinal IgA and IgG ASCs in Peyer’s patches and lamina propria. The reduced ability of serum IgG to bind fecal microbiota despite evidence of increased gut barrier breach is a further indication of the profound reduction in humoral immune responses in Bb-infected mice, despite the paradoxical increased total IgG levels seen. It is consistent also with our previous findings that Bb-infected mice are unable to mount IgG responses to Bb-unrelated antigens (Elsner et al., 2015). More work is needed to determine whether suppression of B cell responses occurs also in human patients, although studies by Robinson and colleagues previously associated strong B cell responses with improved outcomes following Bb infection (Blum et al., 2018).

Secretory IgA (sIgA) is a cornerstone of mucosal defense, essential for neutralizing pathogens, preventing their adherence, and shaping the commensal microbiota through immune exclusion (Corthésy, 2013; Mantis et al., 2011). The marked reduction in sIgA-coated luminal bacteria in Bb-infected mice, despite unchanged total serum IgA, directly reflects the reduced production of local IgA in lamina propria and Peyer’s patches. It has been suggested recently that the levels of IgA binding to the microbiota are a more accurate correlate of host immune status than traditional relative abundance measures of microbial community composition (Olm et al., 2025), further supporting the conclusion that Bb infection has profound effects on the overall immune status of the infected host. The differing kinetics between the early decline in IgA-secreting plasma cells and the later measurable decreases in sIgA-coated bacteria might reflect at least in part the proteolytic resistance of sIgA in the gut lumen (Lindh, 1975). Compromised mucosal IgA responses are reminiscent of findings in autoimmune conditions like celiac disease or inflammatory bowel diseases, where defects in gut barrier integrity and local immune regulation, including IgA production, are implicated in disease pathogenesis (Bamias et al., 2023; Chow et al., 2012).

The profound disruption in local IgA production and reduced coating of the microbiota likely play a pivotal role in the observed ongoing “leakiness” of the GI tract and the ensuing microbial changes. Without sufficient IgA-mediated control, the delicate balance of the luminal microbiota can be disturbed, potentially allowing certain commensal bacteria to translocate across the compromised epithelial barrier, contributing to chronic low-grade endotoxemia. Indeed, we observed significant alterations in the α diversity and compositional shifts in the gut microbial communities of Bb-infected mice, including changes in Bacteroidota, Firmicutes, Verrucomicrobiota, and Actinobacteriota. Consistent with these microbial shifts, we also observed significant changes in the levels of SCFAs, specifically acetic acid in fecal matter. These changes require further analysis as SCFAs have been shown to strongly affect Bb (Lin et al., 2018) itself, but also the host, where shifts in SCFA and gut microbiota composition are increasingly recognized as contributors to the pathogenesis of various systemic and localized diseases, including metabolic disorders, allergies, and autoimmune conditions (Pires et al., 2024). Whether the changes to the microbiota cause changes to the mouse metabolome, developing favorable growth and survival conditions for Bb, is an important question raised by the results from this study.

Our unexpected and disappointing findings regarding the failure of hygromycin A to clear Bb long-term suggest that the evaluation of any antibiotic for use against Bb must be rigorously tested for both short-term and long-term effectiveness, including in mouse models. While we used treatment doses and regimens published for their effectiveness against spirochetes in vitro and short-term in vivo (Leimer et al., 2021), it remains possible that increased doses and/or treatment duration will have a more positive treatment outcome. As expected, ceftriaxone effectively cleared Bb. Because ceftriaxone is a broad-spectrum antibiotic that substantially alters the gut microbiota, we could not determine whether microbiome composition was restored following pathogen clearance. Future studies using pathogen-selective clearance strategies together with microbiota-directed approaches, such as fecal microbiota transplantation, will be needed to define the relative contributions of persistent infection and microbiome alterations to intestinal barrier dysfunction. Furthermore, larger study group sizes would be required to conduct in-depth analyses of individual microbial changes in the GI tract of mice and their linkage to functional changes in the mice.

Another limitation of this study is that we were unable to identify the mechanisms that result in the early increases in gut permeability after Bb infection. While we suspect that initial inflammatory changes in the small intestine are responsible, these changes were overall subtle and blocking of key inflammatory cytokines did not reverse gut permeability. Furthermore, the limited number of clinical samples available and a lack of more frequent blood draws may have made us miss key changes in biomarkers of human infection, such as low-grade endotoxemia that we observed in mice. Nonetheless, our studies suggest that increased focus should be directed toward evaluating humoral systemic and mucosal immunity and the gut microbiome of patients suffering from Lyme disease.

## Materials and methods

### Mice and infection

Female C57BL/6J mice, aged 6–8 wk, were purchased from The Jackson Laboratory. Swiss Webster mice were purchased from Taconic Farms. Experimental mice were housed under specific pathogen-free conditions in ventilated filter-top cages with available food and water at the Johns Hopkins Bloomberg School of Public Health or under similar conditions at the University of California, Davis (UC Davis). The animals were maintained on a 12-h day/night light cycle, with standard environmental conditions of 20–26 °C and 30–70% humidity. Gnotobiotic Swiss Webster mice were purchased from Taconic Farms and bred at the UC Davis Genome and Biomedical Sciences Facility in incubators under germ-free conditions with irradiated 2,918 diet containing 18.4% protein and 6% fat (Teklad) (Savage et al., 2024). Both gnotobiotic mice and their controls were infected by intradermal inoculation via syringe with culture-grown Bb cN40 spirochetes (Olsen et al., 2024). For other mouse experiments, infection was done with host-adapted spirochetes as outlined elsewhere (Olsen et al., 2024). Euthanasia was performed by overexposure to CO_2_ followed by cervical dislocation.

All studies were conducted in compliance with protocols approved by the Johns Hopkins University Animal Care and Use Committee or the Institutional Animal Care and Use Committee of UC Davis.

### Human subjects

All human studies were conducted in strict accordance with protocols approved by the Institutional Review Board of Johns Hopkins University School of Medicine, and written informed consent was obtained from all participants prior to study enrollment. The samples analyzed for this study were part of a longitudinal prospective cohort study (Rebman et al., 2021). For the analysis shown here, data included serum from 20 patients with early Lyme disease who had an erythema migrans lesion ≥5 cm. Patients had a baseline blood draw at the time of diagnosis (visit 1) and a second draw 6 months after the end of antibiotic treatment. At visit 1, all participants were seropositive via a two-tier testing protocol and all were antibiotic-naïve. A majority of these individuals (15/20) also presented with multiple erythema migrans at their initial visit. The 20 Lyme disease patients were matched based on age and gender to 20 control subjects without a clinical history of Lyme disease who were all seronegative at the time of the draw. Both patients and controls were also excluded for a range of comorbid conditions, as previously described (Rebman et al., 2021). Demographic information by group is provided in Table S2.

### Histology

Mice were evaluated at days 3 (*n* = 4), 7 (*n* = 5), 14 (*n* = 5), and 28 (*n* = 5) following Bb infection and were compared against uninoculated animals (*n* = 3) and sham-infected animals at days 14 (*n* = 5) and 28 (*n* = 5). The small intestine was collected from proximal duodenum to distal jejunum, infused gently with 4% paraformaldehyde (PFA), and rolled from distal to proximal in a tissue cassette. The cecum was infused with 4% PFA. The colon was prepared by opening the lumen using fine-tipped scissors, gently removing intact fecal pellets using forceps, and then rolling the tissue from distal to proximal, with the mucosa on the interior, in a tissue cassette. Tissues were then fixed in 4% PFA for 24 h and then transferred into 70% ethanol until paraffin embedding and sectioning performed by the Department of Molecular and Comparative Pathobiology. Briefly, each paraffin block was cut through ∼1/3 depth to ensure that the full length of the mucosa would be visualized in the section. Slides were stained with hematoxylin and eosin. Slides were evaluated at 4× magnification for adequate section and staining quality, and then, one slide per mouse was selected for evaluation. Slides were randomly assigned numbers to achieve blinding of the groups, prior to performing histopathological evaluation using a scoring rubric (Table S1) developed as a modification published in Erben et al. (2014). For MALT evaluation, slides were scanned fully to identify tissue containing MALT for analysis. For other changes, at least 5 longitudinal crypts were evaluated, at the magnifications listed in Table S1. Following scoring, data were unblinded, and average scores were calculated for each scoring category.

### CBC analyses

Analysis of mouse CBCs was done by the UC Davis Comparative Pathology Services. For that, whole blood was collected via tail vein bleeds of infected and sham-infected mice into collection tubes containing EDTA (Microtainer, Becton Dickinson). Samples were placed on ice and then transmitted to the core facility where they were analyzed fresh using an automated hemocytometer. Data were compared with a standard range established for mouse blood samples.

### Antibiotic treatment

For antibiotic treatment, groups of C57BL/6 mice (*n* = 5) were Bb-treated 21 dpi; a third of the Bb-infected group was treated with hygromycin A (200 mg/kg, i.p. for 5 days following previously published protocols but using twice the dose used in the original study; Leimer et al., 2021). Hygromycin A was commercially obtained (Sigma-Aldrich), or, for a separate, independent study, was kindly provided by Dr. Kim Lewis (Northeastern University, Boston, MA, USA). Experimental outcomes were similar between the two batches of hygromycin A. A second group of mice was treated with ceftriaxone (100 mg/kg, i.p. for 30 days; Apotex Corp.), and a third was mock-treated with PBS. All treatments were by i.p. injection. Several tissues were collected to determine the Bb load at 210 dpi, thus about 5 months after completion of antibiotic therapy.

### Quantitative PCR for detection of Bb DNA

Quantitative real-time PCR (qRT-PCR) was performed to detect Bb (*flaB*) DNA, using specific primers and a probe for *flab* (Olsen et al., 2024). Amplification was carried out for 40 cycles under the following conditions: denaturation at 95°C for 15 s, annealing at 50°C for 2 min, and extension at 60°C for 1 min. Tissue samples were weighed, and DNA was extracted using DNeasy kits (Qiagen) according to the manufacturer’s protocol. Purified N40 DNA was used as a positive control, while DNA from uninfected mice and water served as negative controls. Results were expressed as *flaB* DNA copy number per milligram of tissue, calculated using a plasmid standard curve.

### Tissue preparation and immunofluorescence staining

C57BL/6 mice were euthanized and perfused with ice-cold 1× PBS until the effluent ran clear, followed by perfusion with 4% PFA in 1× PBS. Tissues were harvested and postfixed overnight at 4°C in 4% PFA. After several washes in 1× PBS, samples were cryoprotected by immersion in 30% sucrose in 1× PBS overnight at 4°C. The following day, tissues were embedded in Tissue-Tek OCT compound (cat. no. 4583; Sakura) and rapidly frozen on dry ice. Frozen tissues were sectioned to a thickness of 12 μm using a cryostat (cat. no. CM1850; Leica) and mounted on glass slides. For E-cadherin staining, sections were treated with HistoVT One (Nacalai Tesque) at 70°C for 30 min, followed by cooling to room temperature (RT) in 1× PBS for 10 min. All tissue sections were washed in 1× PBS and then blocked for 1 h at RT in blocking buffer containing 10% goat serum (Invitrogen) and 0.5% Triton X-100. Sections were incubated overnight at 4°C with the following primary antibodies diluted in blocking buffer: rat CoraLite Plus 488 anti-mouse CD45 (Proteintech) and rabbit monoclonal E-cadherin (clone 24E10, Cell Signaling Technology). The next day, slides were washed three times with 1× PBS, and sections stained for E-cadherin were incubated with Alexa Fluor 546–conjugated goat anti-rabbit secondary antibody (Invitrogen) for 1 h at RT. All sections were counterstained with DAPI (Sigma-Aldrich) and mounted with Aqua-Mount (Epredia). Fluorescence images were acquired with a 20× objective using a Zeiss LSM 780 confocal microscope and processed using FIJI (ImageJ) software.

### qRT-PCR for cytokine genes

Equivalent-sized portions of the small intestine were collected, and total RNA was extracted using RNeasy Mini Kit (QIAGEN) according to the manufacturer’s instructions. Complementary DNA was synthesized using standard reverse transcription protocols. qRT-PCR was performed using gene-specific commercial primers (Thermo Fisher Scientific, Table S3) and GAPDH as an internal control. Reactions were run on an Applied Biosystems QuantStudio 5 or QuantStudio 6 Flex Real-Time PCR System, and data were analyzed with QuantStudio Real-Time PCR software (Applied Biosystems). Relative gene expression was calculated using the ΔΔCt method.

### FITC-dextran assay

Mice were fasted overnight. The following day, a solution of 80 mg/ml FITC-dextran (4 kDa, Sigma-Aldrich) was prepared in sterile 1× PBS. Each mouse received 150 μl of this FITC-dextran solution via gavage. Four hours after gavage, blood samples were collected into serum collection tubes (BD). The collected serum was diluted 1:4 in PBS and transferred to a black, opaque-bottom 96-well plate (Costar). Fluorescence was determined at 530 nm with excitation at 485 nm in a microplate reader SpectraMax M5 plate reader (Molecular Devices). A standard curve was generated using serum from control mice mixed with known concentrations of FITC-dextran to determine FITC-dextran concentrations in the experimental samples.

### Anti-IL-1β and anti-TNF-α antibody treatment

Mice were administered 200 µg of anti-IL-1β (clone B122, cat. no. BE0246; InVivo MAb) or anti-TNF-α (XT3.11, cat. no. BE0058; InVivo MAb) each neutralizing antibody three times per week, beginning on day 0 of Bb infection and continuing until the time of analysis. An identical treatment regimen was administered to sham-infected control mice.

### Serum biomarker measurements

Endotoxin levels in mouse serum samples were quantified using the Pierce Chromogenic Endotoxin Quant Kit (Thermo Fisher Scientific), strictly following the manufacturer’s protocol. LBP levels in mouse and human serum samples were quantified following the manufacturer’s protocols for the mouse and human LBP kit (both from Abcam), respectively. IFABP concentrations in mouse and human serum samples were quantified following the manufacturer’s protocols for the mouse FABP2/IFABP and human FABP2/IFABP immunoassay (both from Bio-Techne), respectively. Human soluble CD14 concentrations were quantified following the manufacturer’s protocols (Bio-Techne).

### Flow cytometry

Single-cell suspensions from mesenteric lymph nodes and Peyer’s patches were prepared using the frosted-slide method, as described previously (Elsner et al., 2015; Hastey et al., 2012). Surface staining was performed for the detection of GC B cells. Cells were first incubated with an anti-FcγR block for 20 min on ice (10 µg anti-CD16/32, clone 2.4G2, in-house generated), followed by staining on ice for 20 min with the antibody cocktail. The optimal antibody dilution for each conjugate was determined prior to use. The antibody cocktail included anti-CD19-BV786 (clone 1D3, cat. no. 563333; BD Horizon), CD45R-BUV661 (clone RA3-6B2, cat. no. 612972; BD Horizon), CD38-PE-CF594 (clone 90, cat. no. 102730; BioLegend), and CD24-BV711 (clone M1/69, cat. no. 563450; BD Biosciences). A “dump” channel was used to identify and exclude non-B cells from the analysis using the following mAbs conjugated to Pacific Blue: anti-mouse CD90.2 (clone 30-H12, cat. no. 105324; BioLegend), CD4 (clone GK1.5, made in-house), CD8 (clone 53-6.7, cat. no. 100725; BioLegend), NK1.1 (clone PK136, cat. no. 108722; BioLegend), CD11b (clone M1/70, cat. no. 101223; BioLegend), and F4/80 (clone BM-8, made in-house). For dead cell discrimination, cells were stained with Live/Dead Fixable Near-IR (L34994; Thermo Fisher Scientific) on ice for 20 min. For the immunophenotyping of lamina propria cells, samples were similarly incubated with an anti-FcγR block prior to surface staining for CD45-BUV395 (clone 30-F11, cat. no. 564279; BD Horizon), CD3-FITC (clone 145-2C11, cat. no. 100306; BioLegend), CD4-APC-Cy7 (clone GK1.5, cat. no. 552051; BD Biosciences), CD11c-BUV737 (clone N418, cat. no. 367-0114-82; Invitrogen), CD19-BUV661 (clone 1D3, cat. no. 612971; BD Horizon), F4/80-BV421 (clone BM8, cat. no. 123132; BioLegend), CD206-APC (clone C068C2, cat. no. 141704; BioLegend), CD138-PE (clone 281-2, cat. no. 142503; BioLegend), and CD11b-BV605 (clone M1/70, cat. no. 101257; BioLegend). Following surface labeling, the cells were fixed and permeabilized using a Foxp3 buffer for intracellular staining for Foxp3-PE-Cy5 (clone FJK-16s, cat. no. 15-5773-82; Invitrogen) and RORγt- PE-CF594 (clone Q31-378, cat. no. 562664; BD Horizon). Dead cells were separated out using Live/Dead Fixable Aqua (cat. no. L34967; Thermo Fisher Scientific). All flow cytometric data were acquired on a BD Symphony A3 flow cytometer and subsequently analyzed using FlowJo software (version 10.10.0). All flow cytometric data were acquired on a BD Symphony A3 flow cytometer and subsequently analyzed using FlowJo software (version 10.10.0).

### Flow cytometric analysis of IgA- and IgG-coated fecal bacteria

Fecal pellets were homogenized in sterile 1× PBS and centrifuged at 800 *g* for 5 min to remove large debris. The supernatant was collected and centrifuged at 9,200 *g* for 10 min to pellet the fecal bacteria. The bacterial pellet was resuspended in 250 μl FACS buffer, and 50 μl of the suspension was incubated for 30 min at 4°C with anti-mouse IgA-APC (clone mA-6E1, Invitrogen) or anti-mouse IgG-biotin (SouthernBiotech) together with SYBR Green I nucleic acid stain (Invitrogen). For IgG-coated bacteria, streptavidin–APC (BD Pharmingen) was added for 30 min at 4°C following incubation with IgG–biotin and SYBR Green I. Next, samples were washed twice with FACS buffer, fixed with BD Fixation Buffer (BD Biosciences) for 20 min at 4°C, and washed twice. Data were acquired on a BD Symphony A3 flow cytometer by collecting 50,000 events per sample at a low flow rate. Data were analyzed using FlowJo software (version 10.10.0).

### IgA binding assay with fecal bacteria

Fecal bacteria were collected as described in the preceding section. The serum from the same mouse was diluted 1:10 in 1× PBS and incubated with the pelleted bacteria at 37°C for 1 h in 100 μl. Following incubation, the bacteria were washed once with FACS buffer. Staining and flow cytometric analysis were performed as described in the previous section.

### Lamina propria lymphocyte isolation

Lamina propria lymphocytes were isolated from the ileum or colon. Briefly, tissue was collected and flushed with ice-cold 1× HBSS to remove fecal matter. Fat and Peyer’s patches were removed, and the tissue was longitudinally opened and washed in ice-cold 2% FBS/HBSS. The tissue was then cut into 1-cm segments and washed in 1× HBSS. Epithelial cells were removed by two sequential incubations in 2 mM EDTA/HBSS at 37°C for 30 and 15 min, respectively, with shaking at 250 RPM. The remaining tissue was digested in an enzyme cocktail containing 0.1% collagenase I, 0.25% collagenase IV, Dispase (0.25 U/ml), and DNase I (7.5 μg/ml) in complete RPMI at 37°C for 30 min while shaking at 250 rpm. The cell suspension was then filtered through 100-μm strainers. In some experiments, the cell pellet was further purified using a Percoll density (Cytiva) gradient to isolate the leukocyte fraction. Cells were washed twice by centrifugation at 1,500 rpm for 10 min in RPMI media and resuspended in complete RPMI for further analysis.

### ELISpot assay for IgA- and IgG-secreting cells

MultiScreen-HA filtration plates (cat. no. MAHAS4510; Millipore) were coated overnight at RT with anti-mouse IgA or anti-mouse IgG (in 1× PBS [100 μl per well]) in a humidified chamber. Plates were washed sequentially with 1× PBS containing 0.05% Tween-20, 1× PBS, and deionized water, then blocked with 1× PBS containing 4% BSA (100 μl per well) for 1 h at RT. Single-cell suspensions from Peyer’s patches and lamina propria were prepared and serially diluted in complete RPMI medium (RPMI+ 10% FBS, 100× penicillin–streptomycin, 100× L-glutamine, and 100× 2-mercaptoethanol). Cells (1 × 10^6^ per well, or appropriate serial dilutions in 50 μl) were added to wells and incubated overnight at 37°C in a humidified 5% CO_2_ incubator. The following day, plates were washed and incubated for 2 h at RT with biotinylated anti-mouse IgA or IgG diluted in 1× PBS containing 2% BSA (50 μl per well). Plates were then incubated with streptavidin–HRP for 1 h at RT. Following additional washes, a freshly prepared 3-amino-9-ethylcarbazole (AEC) substrate solution (20 mg AEC dissolved in N,N-dimethylformamide, added to 0.1 M sodium acetate, pH 5.0, and filtered, with 15 ml of 30% H_2_O_2_ added immediately before use) was added (100 μl per well) and incubated for 20 min at RT. The reaction was stopped by rinsing with tap water, and plates were air-dried. Spots were enumerated and analyzed using an AID ELISpot reader.

### ELISA for total IgG and IgA

ELISAs were performed using MaxiSorp 96-well microplates (427; Thermo Fisher Scientific). Plates were coated overnight at 4°C with anti-mouse IgG or IgA antibodies (SouthernBiotech) in 1× PBS. Plates were washed (described in the previous section) and blocked for 1 h at RT with ELISA blocking buffer (1% new born calf serum, 0.1% dried milk powder, 0.05% Tween-20 in 1× PBS). Serum samples were serially diluted and added to the plates, followed by incubation for 2 h at RT. Plates were washed and incubated for 1 h with biotin-conjugated anti-mouse IgG or anti-mouse IgA (SouthernBiotech), followed by streptavidin–HRP (Vector Laboratories) for 1 h. Standard curves were generated using purified mouse IgG or IgA (SouthernBiotech). A substrate solution was added, reactions were stopped with 1 N sulfuric acid, and absorbance was measured at 450 nm with a reference wavelength of 595 nm using a SpectraMax M5 plate reader (Molecular Devices).

### 16S rRNA sequencing and analysis

Fecal pellets were collected at various time points and stored at −80°C prior to shipment to Microbiome Insights for DNA processing and sequencing. Briefly, DNA was extracted from fecal pellets using MoBio PowerMag Soil DNA Isolation Bead Plate on a KingFisher robot. The V4 region of the bacterial 16S rRNA gene was amplified by PCR using dual-barcoded primers (515F and 806R) (Kozich et al., 2013). Amplicons were sequenced on an Illumina MiSeq using the 300-bp paired-end kit (version 3). Sequences were processed using the mothur software package (version 1.44.1) following the MiSeq SOP, including denoising, taxonomic classification with the Silva (version 138) database, and clustering into 97% similarity OTUs (Data S1). To quantify OTU-level changes across groups and time points, read abundance for each OTU was first calculated per sample and then normalized by expressing each OTU relative to the mean abundance of the corresponding OTU in sham-infected control mice. This approach allowed visualization of infection-associated directional changes in bacterial taxa over time. To address potential contamination, template-free controls and extraction kit reagents were cosequenced. OTUs were considered contaminants and removed if their mean abundance in controls exceeded 25% of their abundance in specimens. Alpha diversity was measured using the Shannon index on contaminant-filtered abundance tables, and differences were tested with ANOVA or a linear mixed model. For β diversity, OTUs with a count of less than three in at least 10% of samples were excluded before computing Bray–Curtis indices. Community structure was visualized with principal coordinate analysis and assessed with permutational multivariate analyses of variance using 999 permutations. All analyses were conducted in R.

### SCFA isolation and analyses

Fecal pellets were resuspended in Milli-Q water-grade H_2_O and homogenized using MP Bio FastPrep for 1 min at 4.0 m/s. 5 M HCl was added to acidify fecal suspensions to a final pH of 2.0. Acidified fecal suspensions were incubated and centrifuged at 10,000 rpm to separate the supernatant. Fecal supernatants were spiked with 2-ethylbutyric acid for a final concentration of 1 mM. Extracted SCFA supernatants were stored in 2-ml GC vials, with glass inserts. SCFAs were detected using gas chromatography (Thermo Trace 1310), coupled to a flame ionization detector (Thermo). The gas chromatography column used was a Thermo TG-WAXMS A (30 m, 0.32 mm, 0.25 µm). Data were analyzed on a gas chromatography FID. Concentrations were normalized to the amount of input material.

### Online supplemental material

Fig. S1 shows the pathological evaluation of the colon and cecum during Bb infection. Fig. S2 shows the phenotypic characterization of lamina propria immune cell populations following Bb infection. Fig. S3 shows the effect of IL-1β and TNF-α neutralization on intestinal permeability during Bb infection. Fig. S4 shows serum biomarkers in Lyme disease patients who returned to health versus those with long-term symptoms. Fig. S5 shows the analysis of Bb tissue burden of mice following antibiotic treatment with hygromycin A and ceftriaxone and sham treatment at day 210 after Bb infection. Table S1 provides the histological scoring rubric. Table S2 provides human demographic data. Table S3 lists the qRT-PCR primers used. Data S1 is related to Fig. 6 and contains detailed analyses of OTU counts at different time points, having pairwise comparisons of Bb-infected mice to sham-infected controls at each time point..

## Supplementary Material

Table S1 shows histological scoring rubric.

Table S2 shows human demographic data.

Table S3 shows qRT-PCR primers used.

Data S1 contains detailed analyses of OTU counts at different time points, having pairwise comparisons of Bb-infected mice to sham-infected controls at each time point.

## Data Availability

16S gut bacterial sequencing data from individual mice are uploaded to the NCBI BioSample Database, accession no. PRJNA1501871, and summaries are provided in Data S1 (related to Fig. 6). Other data are provided in the figures, tables, and supplemental information provided with the manuscript. Additional information will be made available by the corresponding author upon reasonable request.
