# Supplementary material for: Borrelia burgdorferi disrupts gut barrier functions and microbiota–antibody interactions
Source: J Exp Med. 2026 Aug 24;223(9):e20252380. doi: 10.1084/jem.20252380 (PMC13502156; doi:10.1084/jem.20252380)
Supplement: Supplementary file 1 [file jem_20252380_tables1.docx]

**Table S1: Histologic Scoring Rubric**

| **Category** | **Criteria** | **Score** | **Definition** |  | **Magnification assessed** |
| --- | --- | --- | --- | --- | --- |
| Mononuclear infiltrate | Extent | 0 | <10% per hpf |  | 40X |
|  |  | 1 | 10-25% |  |  |
|  |  | 2 | 26-50% |  |  |
|  |  | 3 | >50% with dense infiltrate |  |  |
|  |  |  |  |  |  |
|  | Severity | 0 | Lamina propria only |  |  |
|  |  | 1 | Mucosa |  |  |
|  |  | 2 | Mucosa and submucosa |  |  |
|  |  | 3 | Mucosa, submucosa, and transmural |  |  |
|  |  |  |  |  |  |
| Neutrophilic infiltrate | Extent | 0 | <10% per hpf |  | 40X |
|  |  | 1 | 10-25% |  |  |
|  |  | 2 | 26-50% |  |  |
|  |  | 3 | >50% with dense infiltrate |  |  |
|  |  |  |  |  |  |
|  | Severity | 0 | Lamina propria only |  |  |
|  |  | 1 | Mucosa |  |  |
|  |  | 2 | Mucosa and submucosa |  |  |
|  |  | 3 | Mucosa, submucosa, and transmural |  |  |
|  |  |  |  |  |  |
| Epithelial changes* | Crypt hyperplasia | 0 | <10% |  | 40X |
|  |  | 1 | <25% |  |  |
|  |  | 2 | 25-35% |  |  |
|  |  | 3 | 36-50% with mitoses in middle/upper third of crypt epithelium, distant from crypt base |  |  |
|  |  | 4 | >51% with mitoses in upper third of crypt epithelium, distant from crypt base |  |  |
|  |  |  |  |  |  |
|  | Goblet cell loss | 0 | <10% |  | 40X |
|  |  | 1 | 11-20% |  |  |
|  |  | 2 | 21-35% |  |  |
|  |  | 3 | 36-50% |  |  |
|  |  | 4 | >50% |  |  |
|  |  |  |  |  |  |
|  | Erosion (loss of surface epithelium with intact basement membrane) | 0 | Absent |  | 10-40X |
|  |  | 1 | Present - describe (e.g. local, diffuse, etc.) |  |  |
|  |  |  |  |  |  |
|  |  |  |  |  |  |
|  |  |  |  |  |  |
|  |  | | |  |  |
|  | Ulceration (epithelial defect that reaches beyond the muscularis mucosae) | 0 | Absent |  | 10-40X |
|  |  | 1 | Present - describe (e.g. local, diffuse, etc.) |  |  |
|  |  |  |  |  |  |
|  |  |  |  |  |  |
|  |  |  |  |  |  |
|  |  |  |  |  |  |
| Mucosal architecture* | Structural changes to crypts | 0 | Absent |  | 40X |
|  |  | 1 | Structural changes present (e.g. irregular crypts, non-parallel, variable crypt diameters) |  |  |
|  |  | 2 | Crypt loss |  |  |
|  |  |  |  |  |  |
|  | Villous blunting (SI only) | 0 | Absent |  |  |
|  |  | 1 | Mild |  |  |
|  |  | 2 | Moderate |  |  |
|  |  | 3 | Severe / villous atrophy |  |  |
|  |  |  |  |  |  |
| Paneth cell degeneration (SI) | Paneth cell vacuolization | 0 | Absent |  | 40X |
|  |  | 1 | Mild |  |  |
|  |  | 2 | Moderate |  |  |
|  |  | 3 | Marked |  |  |
|  |  |  |  |  |  |
|  | Granule size pleiomorphism | 0 | Absent |  |  |
|  |  | 1 | Mild |  |  |
|  |  | 2 | Moderate |  |  |
|  |  | 3 | Marked |  |  |
|  |  |  |  |  |  |
| MALT (SI) | Size of MALT | 0 | Normal |  | 4X |
|  |  | 1 | Mild enlargement |  |  |
|  |  | 2 | Moderate enlargement |  |  |
|  |  | 3 | Marked enlargement |  |  |
|  |  |  |  |  |  |

* Evaluated at least 5 longitudinally oriented crypts
