# Supplementary material for: Borrelia burgdorferi disrupts gut barrier functions and microbiota–antibody interactions
Source: J Exp Med. 2026 Aug 24;223(9):e20252380. doi: 10.1084/jem.20252380 (PMC13502156; doi:10.1084/jem.20252380)
Supplement: Supplementary file 2 [file jem_20252380_tables2.docx]

**Table S2: Human Demographic Data**

|  | **Early Lyme Disease**  (n=20) | **Controls**  (n=20) | ***p*-value** |
| --- | --- | --- | --- |
| **Age (years)**  Median, (Range) | 56.0, (25.0-78.0) | 58.5, (23.0-80.0) | 0.87 |
| **Gender**  Male (%)  Female (%) | 55.0  45.0 | 55.0  45.0 | 1.00 |
| **Race/Ethnicity**  White (%)  Hispanic (%)  Other (%) | 100.0  0.0  0.0 | 85.0  5.0  10.0 | 0.23  -  - |
| **Signs of disease**  Multiple Erythema (%)  Single Erythema (%) | 80.0  20.0 | N/A | N/A |
| **Diagnosis**  Two-Tier Antibody Test (%) | 100.0 | 0.0 | <0.0001 |
| **Disease duration at the time of sample collection (days)**  Median, (Range) | 7.0, (3.0-60.0) | N/A | N/A |
| **Blood Cell Count**  Neutrophils  Lymphocytes  Ratio (Neutrophils/Lymphocytes)  Median, (Range) | 5.47, (1.88-10.65)  1.36, (0.64-1.94)  4.04, (1.54-8.59) | 3.43, (1.64-5.66)  1.75, (0.52-2.94)  2.03, (0.67-8.11) | 0.006  0.016  0.0002 |
| **Liver functions tests**  Aspartate Aminotransferase (AST)  Alanine transaminase (ALT)  Alkaline phosphatase (ALK)  Median, (Range) | 24.0 (15.0-78.0)  44.0 (15.0-140.0)  92.5 (29.0-293.0) | 20.0 (13.0-35.0)  20.0 (8.0-43.0)  59.0 (41.0-109.0) | 0.025  0.001  0.0006 |
| **Number of acute-onset Lyme disease symptoms (in addition to EM rash)** | 11.0 (0.0-29.0) | N/A | N/A |

N(%) for categorical and median [IQR] (range) for continuous because duration of illness was not normally distributed.

p-values were calculated via Fisher’s exact test for categorical and Wilcoxon rank sum test for continuous variables.
