# Supplementary material for: Borrelia burgdorferi disrupts gut barrier functions and microbiota–antibody interactions
Source: J Exp Med. 2026 Aug 24;223(9):e20252380. doi: 10.1084/jem.20252380 (PMC13502156; doi:10.1084/jem.20252380)
Supplement: Supplementary file 3 [file jem_20252380_tables3.docx]

**Table S3: qRT-PCR Primers Used**

| **Gene** | **Species** | **Catalog Number** |
| --- | --- | --- |
| *IL6* | *Mus musculus* | *Mm00446190_m1* |
| *IL10* | *Mus musculus* | *Mm00439616_m1* |
| *Ocln* | *Mus musculus* | *Mm00500910_m1* |
| *Ifng* | *Mus musculus* | *Mm01168134_m1* |
| *Tjp1* | *Mus musculus* | *Mm01320638_m1* |
| *Il1β* | *Mus musculus* | *Mm00434228_m1* |
| *Cdh1* | *Mus musculus* | *Mm01247357_m1* |
| *Tnfa* | *Mus musculus* | *Mm00443258_m1* |
| *Gapdh* | *Mus musculus* | *Mm99999915_g1* |
